# Novel phenothiazine-based sensitizers for high-performance dye-sensitized solar cells: enhanced photovoltaic properties through strategic Co-sensitization with N719†

**DOI:** 10.1039/d5ra00694e

**Published:** 2025-04-30

**Authors:** Sara H. Yousef, Ehab Abdel-Latif, Safa A. Badawy, Mohamed R. Elmorsy

**Affiliations:** a Department of Chemistry, Faculty of Science, Mansoura University Mansoura 35516 Egypt ehabattia00@gmx.net

## Abstract

This study presents a systematic investigation of novel phenothiazine-based sensitizers (SR1–6) for dye-sensitized solar cells (DSSCs), both as individual sensitizers and in co-sensitization with ruthenium-based N-719 dye. The compounds exhibited notable spectral properties when adsorbed on TiO_2_, demonstrating significant bathochromic shifts and broadened absorption profiles, indicative of strong electronic coupling with the semiconductor surface. Electrochemical characterization confirmed optimal energy level alignment, with ground state oxidation potentials (GSOP) ranging from −5.75 to −6.02 eV and excited state oxidation potentials (ESOP) between −3.54 and −3.77 eV, facilitating efficient electron injection and dye regeneration. In single-dye configurations, SR1 achieved the highest efficiency of 4.22% with a short-circuit current density (*J*_sc_) of 11.96 mA cm^−2^, while co-sensitization with N-719 resulted in substantial improvements, particularly for SR6 + N-719, which attained 9.77% efficiency with a *J*_sc_ of 21.63 mA cm^−2^. Electrochemical impedance spectroscopy revealed that successful co-sensitized devices exhibited enhanced charge transfer resistance (*R*_ct_) values, indicating reduced electron recombination and improved interface stability. This comprehensive study provides valuable insights into molecular design strategies for efficient DSSC sensitizers and demonstrates the efficacy of strategic co-sensitization approaches.

## Abstract 2

This study presents a systematic investigation of novel phenothiazine-based sensitizers (SR1–6) for dye-sensitized solar cells (DSSCs), both as individual sensitizers and in co-sensitization with ruthenium-based N-719 dye.

## Graphical abstract

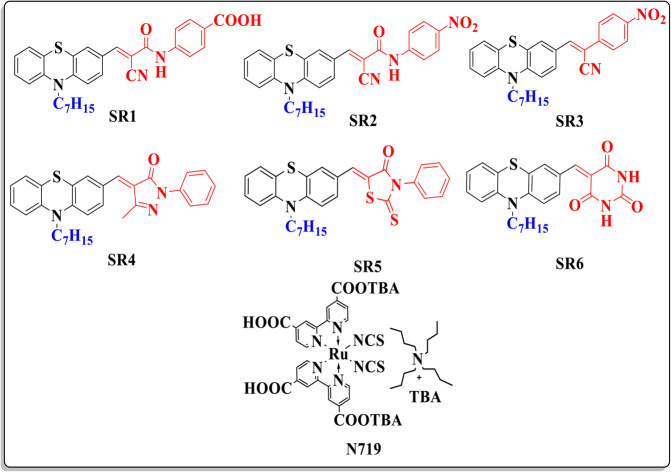

## 1. Introduction

**Fig. 1:**
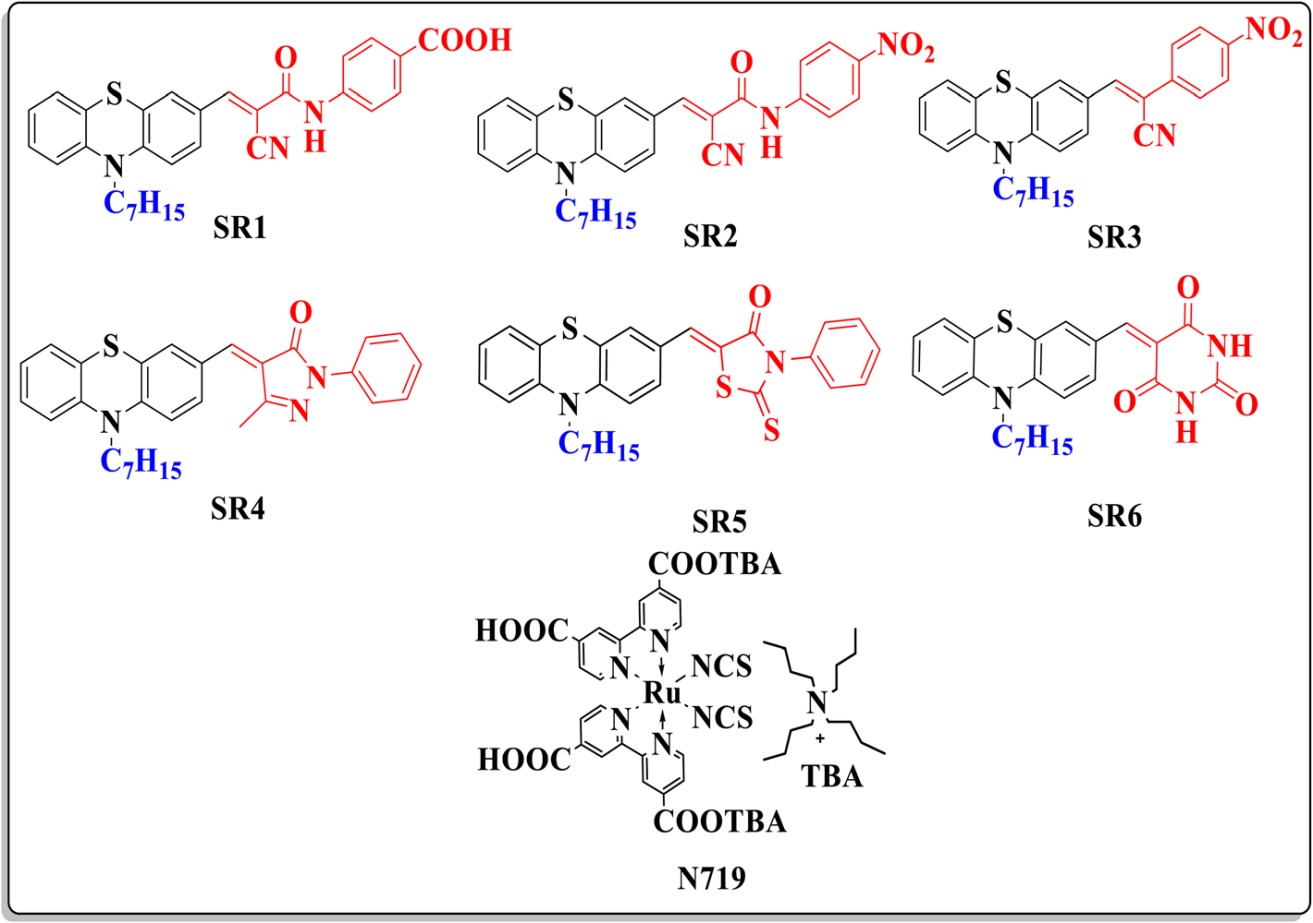
Molecular structures of sensitizers SR1–6 compared to N719.

The escalating global energy demand, coupled with the urgent need to address climate change, underscores the necessity of transitioning from fossil fuels to sustainable energy sources. This challenge is further intensified by population growth and the increasing energy requirements of developing nations. To meet these demands, it is imperative to identify renewable energy solutions that are both cost-efficient and reliant on widely available raw materials. Among the various renewable energy options, solar energy emerges as a particularly promising candidate, offering an abundant and economically viable resource that has long been harnessed by nature to sustain life on Earth. Thus, it seems that the only practical solution to the energy problem on a big scale is to use photovoltaic technologies to harvest the sun's power.^1^ The commercialized PV devices initially generated, which used cells made of silicon, had an efficiency of more than 25%. Nevertheless, the extensive usage of these gadgets is limited by their expensive methods.^2^ Because of this, Organic Photovoltaics, or OPVs, or Organic Solar Cells, have recently attracted a lot of interest from industry and researchers. In 1991, O'Regan and Graztel invented dye-sensitized solar cells (DSSCs), a new type of photosensitizer that is becoming more and more popular as a viable alternative to high-cost conventional silicon solar cells because of inexpensive material cost, structural tunability, and relatively high performance, as well as simple fabrication process.^3^ DSSCs structure consists of a nanocrystalline TiO_2_ photoanode, sensitized with metal complexes or metal-free dye molecules to facilitate light harvesting. This photoanode is immersed in an electrolyte containing the *I*^−^/*I*_3_^−^ redox couple, enabling efficient charge transport and regeneration.^4^ In the past two decades, various types of photosensitizers have been developed, including metal-containing complexes (zinc polypyridine and ruthenium porphyrin) and metal-free dyes.^5^ The potential of ruthenium and zinc metal complex dyes for use in dye-sensitized solar cells has been the subject of substantial research. The molar extinction coefficients of ruthenium dyes are low, though, and the metal itself is expensive and uncommon. Due to their greater molar extinction coefficients, simple synthesis, and significantly lower cost, as well as the fact that their production requires laborious purifying methods, pure organic dyes have received more attention in the field of research and development.^6^ Significant advancements have been made in this field, and a number of potential electron donors like triphenylamine, diphenylamine, carbazole, indoline, tetrahydroquinoline, phenoxazine, and phenothiazine have been investigated.^7^ Among many kinds of metal-free organic sensitizers, the phenothiazine (PTZ)-based organic dyes have garnered significant scientific attention since their initial investigation by Sun *et al.* in 2007.^8^ The introduction of PTZ as a donor in the molecular structure of the sensitizer has resulted in photovoltaic performance that meets acceptable standards. Because of its ground-state non-planar butterfly shape, which reduces aggregation, PTZ donors are an especially promising kind of donor.^9^ Moreover, PTZ is a heterocyclic molecule containing a nitrogen atom and an electron-rich sulfur atom in the same six-membered ring, making it an extremely potent electron donor, surpassing the capabilities of triphenylamine, tetrahydroquinoline, carbazole, iminodibenzyles and other *N*-heterocycles.^10^ In light of this, we developed and produced a class of chemical dyes (SR1–6) in this study that have phenothiazine as a potent donor moiety linked to various acceptor moieties (Fig. 1). Furthermore, the experimental data was verified by theoretical calculations based on density functional theory carried out by Gaussian software. The choice of these six acceptor moieties for phenothiazine-based sensitizers (SR1–SR6) was driven by their potential to enhance intramolecular charge transfer (ICT), optimize energy level alignment, and improve light absorption in (DSSCs). These acceptors 4-cyanoacetamide derivatives (SR1–2), nitroacetonitrile (SR3), pyrazolone (SR4), thiazolidinone (SR5), and barbituric acid (SR6) were carefully selected to explore the effect of different electron-withdrawing groups on DSSC performance.^10,11^ Compared to state-of-the-art acceptor units such as cyanoacrylic acid, and pyridine derivatives, these moieties offer stronger electronic coupling with the TiO_2_ surface, extended conjugation, and enhanced dye adsorption, all of which are critical for efficient electron injection and reduced charge recombination.^11^ Notably, barbituric acid (SR6) and thiazolidinone (SR5) have been previously explored in different dye backbones, but their integration into phenothiazine-based sensitizers is relatively novel, allowing for enhanced charge separation due to the non-planar butterfly structure of phenothiazine. Similarly, pyrazolone (SR4) has been widely utilized in organic electronics, but its potential in DSSCs remains underexplored, making it a promising candidate for improving light absorption while minimizing dye aggregation. The cyanoacetamide (SR1) and cyanoacetanilide (SR2) groups, on the other hand, are well-known for their strong electron-withdrawing nature, yet their precise influence on DSSC performance when attached to a phenothiazine donor has not been extensively studied. By systematically varying these acceptor moieties, this study provides new insights into structure–property relationships, demonstrating how different electron-withdrawing units impact DSSC efficiency, charge transfer resistance, and spectral absorption. This approach allows for a more rational molecular design strategy compared to traditional DSSC sensitizers, potentially paving the way for higher efficiency, lower recombination losses, and broader light absorption spectra in future dye engineering.

## 2. Experimental section

### 2.1. Synthesis

#### 2.1.1. Synthesis of 10-heptyl-10*H*-phenothiazine-3-carbaldehyde (3)

10-Heptyl-10*H*-phenothiazine-3-carbaldehyde (3) has been synthesized through two reactions. Firstly, alkylation reaction between (0.2 g, 1 mmol phenothiazine) and (0.3 mL heptyl bromide) with (0.3 g NaH) in presence of DMF, then, stirring for 2 h. Poured into 100 mL ice-cold water and extracted with ethyl acetate. The oily heptyl phenothiazine compound is produced. Subsequently, 2.8 mL (30 mmol) of newly purified POCl_3_ was added dropwise to a stirred solution of dry DMF (2.75 mL, 35 mmol) at 0 °C under an argon atmosphere until the colored vilsmeier salt fully precipitated. A solution of 10-heptyl-10*H*-phenothiazine (0.3 g, 1 mmol) in 10 mL of DMF was added to the reaction mixture dropwise while continuously stirring for 1 hour. The temperature was elevated to 70 °C, and thereafter, the reaction mixture was stirred overnight. Thereafter, the mixture was placed into 100 mL of ice-cold water and extracted using ethyl acetate. The oily 10-heptyl-10*H*-phenothiazine-3-carbaldehyde product is formed^12^ with yield 93%. The specific SR1–6 sensitizers were produced by means of a Knoevenagel reaction.

#### 2.1.2. General synthesis of sensitizers SR1–6

10-Heptyl-10*H*-phenothiazine-3-carbaldehyde (3) (0.32 g, 1 mmol) was dissolved in 20 mL acetic acid added to 1 mmol of corresponding sensitizer (namely,4-(2-cyanoacetamido)benzoic acid (4a), 2-cyano-*N*-(4-nitrophenyl)acetamide (4b), 2-(4-nitrophenyl)acetonitrile (5), 5-methyl-2-phenyl-2,4-dihydro-3*H*-pyrazol-3-one (6), 3-phenyl-2-thioxothiazolidin-4-one (7) and pyrimidine-2,4,6 (1*H*,3*H*,5*H*)-trione) (8) then (0.1 g, 1 mmol) of sodium acetate in a round-bottomed flask. The flask's contents were heated for 5 hours, then cooled to room temperature, resulting in the formation of a precipitate. Subsequently, the precipitate was purified by recrystallization from ethanol. Fig. from S2 to S25 †showed all the spectral analysis including (IR, ^1^HNMR, ^13^CNMR and mass) for compounds SR1–6.

#### 2.1.3. 4-(2-Cyano-3-(10-heptyl-10*H*-phenothiazin-3-yl) acrylamido) benzoic acid (SR1)

Red crystal (78% yield); m.p. = 230–232 °C. IR (KBr) *ν*_max_ cm^−1^: 3330 (N–H), 3014–2834 (C–H aliphatic), 2212 (C≡N), 1675 (C=O). ^1^H NMR (DMSO*-d*_6_): *δ* 0.83 (br. s, 3H, CH_3_), 1.23 (s, 6H, CH_2_), 1.40 (s, 2H, CH_2_), 1.71 (s, 2H, CH_2_), 3.96 (s, 2H, CH_2_), 7.02–7.03 (m, 1H, Ar–H), 7.10 (d, *J* = 7.20 Hz, 1H, Ar–H), 7.18–7.24 (m, 3H, Ar–H), 7.81 (d, *J* = 7.20 Hz, 3H, Ar–H), 7.89 (d, *J* = 7.60, 1H, Ar–H), 7.96 (d, *J* = 7.20 Hz, 2H, Ar–H), 8.16 (s, 1H, =CH), 10.57 (s, 1H, N–H), 12.83 (s, 1H, OH) ppm. ^13^C NMR (DMSO-*d*_6_): *δ* 14.39, 22.42, 26.43, 26.54, 28.68, 31.68, 47.35, 103.51, 116.27, 116.96, 117.22, 120.19, 122.59, 123.77, 124.10, 126.20, 126.44, 126.49, 127.78, 128.51, 128.99, 130.76 (2C), 131.65, 142.96, 143.26, 149.04, 150.22, 161.77, 167,34 ppm. Mass analysis (*m*/*z*, %): 511 (M^+^, 31.15), 510 (100.00), 508 (57.35), 340 (76.98), 329 (67.90), 281 (78.93), 138 (48.67), 85 (56.99), 80 (63.67). Analysis for C_30_H_29_N_3_O_3_S (511.64): calculated: C, 70.43; H, 5.71; N, 8.21%. Found: C, 70.54; H, 5.77; N, 8.14%.

#### 2.1.4. 2-Cyano-3-(10-heptyl-10*H*-phenothiazin-3-yl)-*N*-(4-nitrophenyl) acrylamide (SR2)

Dark red crystal (74% yield); m.p. = 200–202 °C. IR (KBr) *ν*_max_ cm^−1^: 3326 (N–H), 2920–2847 (C–H aliphatic), 2207 (C≡N), 1682 cm^−1^ (C=O) cm^−1^. ^1^H NMR (DMSO-*d*_6_): *δ* 0.79–0.82 (m, 3H, CH_3_), 1.21–1.23 (m, 4H, CH_2_), 1.25–1.28 (m, 2H, CH_2_), 1.36–1.39 (m, 2H, CH_2_), 1.67–1.70 (m, 2H, CH_2_), 3.94 (t, *J* = 7.50 Hz, 2H, CH_2_), 7.00 (t, *J* = 8.00 Hz, 1H, Ar–H), 7.08 (d, *J* = 8.00 Hz, 1H, Ar–H), 7.15–7.24 (m, 3H, Ar–H),7.78 (s, 1H, Ar–H), 7,88 (d, *J* = 8.50 Hz, 1H, Ar–H), 7.92 (d, *J* = 8.50 Hz, 2H, Ar–H), 8.169 (s, 1H, =CH), 8.26 (d, *J* = 8.50 Hz, 2H, Ar–H), 10.80 (s, 1H, NH) ppm. ^13^C NMR (DMSO-*d*_6_): *δ*13.91, 21.94, 25.94 (2C), 28.19, 31.21, 46.90, 102.65, 115.82, 116.54, 116.62, 120.15 (2C), 122.08, 123.30, 123.69, 124.84 (2C), 125.60, 127.32, 128.07, 128.59, 131.37, 142.72, 142.83, 144.68, 148.81, 150.288, 161.69 ppm. Mass analysis (*m*/*z*, %): 512 (M^+^, 90.73), 488 (53.25), 445 (59.90), 378 (51.65), 351 (77.18), 345 (51.99), 271 (78.35), 201 (100.00), 156 (52.23), 138 (51.41), 136 (57.62), 73 (62.86). Analysis for C_29_H_28_N_4_O_3_S (512.63): calculated: C, 67.95; H, 5.51; N, 10.93%. Found: C, 68.09, H, 5.58, N, 10.83%.

#### 2.1.5. 3-(10-Heptyl-10*H*-phenothiazin-3-yl)-2-(4-nitrophenyl) acrylonitrile (SR3)

Red solid (62% yield); m.p. = 220–222 °C. IR (KBr) *ν*_max_ cm^−1^: 2919–2849 (C–H aliphatic), 2210 (C≡N) cm^−1^;^1^H NMR (DMSO*-d*_6_): *δ* 0.83 (t, *J* = 7.20 Hz, 3H, CH3), 1.23–1.30 (m, 6H, CH_2_), 1.38–1.42 (m, 2H,CH_2_), 1.68–1.72 (m, 2H, CH_2_), 3.94 (t, *J* = 6.80 Hz, 2H, CH_2_), 7.01 (t, *J* = 7.60 Hz, 1H, Ar–H), 7.08 (d, *J* = 8.40 Hz, 1H, Ar–H), 7.17–7.20 (m,2H, Ar–H), 7.24 (t, *J* = 8.00 Hz, 1H, Ar–H), 7.80 (s, 1H, Ar–H), 7.90 (d, *J* = 8.80 Hz, 1H, Ar–H), 7.99 (d, *J* = 8.80 Hz, 2H, Ar–H), 8.15 (s, 1H, =CH), 8.34 (d, *J* = 8.80 Hz, 2H, Ar–H) ppm. ^13^C NMR (DMSO-*d*_6_): *δ* 14.39, 22.42, 26.44, 26.55, 28.68, 31.69, 47.26, 105.22, 116.21, 116.32, 118.32, 122.64, 123.71, 123.89, 124.82 (2C), 126.96 (2C), 127.75 (2C), 128.46, 128.52, 130.65, 141.01, 143.54, 145.22, 147.40, 147.99 ppm. Mass analysis (*m*/*z*, %): 470 (M^+^, 4.96), 449 (69.55), 423 (94.87), 418 (69.19), 400 (60.76), 394 (93.99), 379 (77.00), 370 (68.15), 369 (86.19), 366 (68.38), 336 (80.88), 324 (90.89), 170 (100.00), 129 (67.43), 109 (70.09), 74 (60.35). Analysis for C_28_H_27_N_3_O_2_S (469.60): calculated: C, 71.62; H, 5.80; N, 8.95%. Found: C, 71.73; H, 5.75; N, 9.01%.

#### 2.1.6. 4-((10-Heptyl-10*H*-phenothiazin-3-yl)methylene)-5-methyl-2-phenyl-2,4-dihydro-3*H*-pyrazol-3-one (SR4)

Reddish black crystal (66% yield); m.p. = 184–186 °C. IR (KBr) *ν*_max_ cm^−1^: 2920, 2848 (C–H aliphatic), 1677 (C=O) cm^−1^. ^1^H NMR (DMSO-*d*_6_): *δ* 0.78 (t, *J* = 7.00 Hz, 3H, CH_3_), 1.18–1.25 (m, 6H, CH_2_),1.30–1.34 (m, 2H, CH_2_), 1.60–1.64 (m, 2H, CH_2_), 2.30 (s, 3H, CH_3_-pyrazolone), 3.78 (t, *J* = 6.00 Hz, 2H, CH_2_), 6.87–6.91 (m, 2H, Ar–H), 6.95–7.00 (m, 2H, Ar–H),7.07 (t, *J* = 9.50 Hz, 2H, Ar–H), 7.14–7.18 (m, 1H, Ar–H), 7.19–7.23 (m, 2H, Ar–H), 7.38–7.47 (m, 2H, 1H, =CH, Ar–H),7.68 (d, *J* = 8.00 Hz, 2H, Ar–H). ^13^C NMR (DMSO-*d*_6_): *δ* 11.86, 13.94, 22.00, 26.21, 28.33, 31.26, 32.58, 46.35, 115.37, 115.54, 116.68, 118.31, 120.31 (2C), 122.20, 122.94, 125.21, 125.76, 126.49, 127.10, 127.56, 128.86 (3C), 137.20, 137.85, 142.74, 144.87, 146.11, 161.93 ppm. Mass analysis (*m*/*z*, %): 481 (M^+^, 59.09), 393 (68.73), 382 (66.64), 347 (57.04), 344 (60.16), 321 (100.00), 317 (79.69), 278 (77.81), 238 (61.54), 183 (53.42), 154 (65.69). Analysis for C_30_H_31_N_3_OS (481.66): calculated: C, 74.81; H, 6.49; N, 8.72%. Found: C, 74.64, H, 6.55, N, 8.78%.

#### 2.1.7. 5-((10-Heptyl-10*H*-phenothiazin-3-yl) methylene)-3-phenyl-2-thioxothiazolidin-4-one (SR5)

Dark red crystal (64% yield); m.p. = 130–132 °C. IR (KBr) *ν*_max_ cm^−1^: 2954–2851 (C–H aliphatic), 1704 (C=O) cm^−1^. ^1^H NMR (DMSO-*d*_6_): *δ* 0.03 (t, *J* = 7.00 Hz, 3H, CH_3_), 0.37–0.42 (m, 6H, CH_2_), 0.52–0.54 (m, 2H, CH_2_), 0.83–0.85 (m, 2H, CH_2_), 3.07 (t, *J* = 6.50 Hz, 2H, CH_2_), 6.14 (t, *J* = 7.50 Hz, 1H, Ar–H), 6.22 (d, *J* = 8.00 Hz, 1H, Ar–H),6.32 (t, *J* = 6.50 Hz, 2H, Ar–H),6.37 (t, *J* = 7.50 Hz, 1H, Ar–H), 6.54 (d, *J* = 8.00 Hz, 2H, Ar–H), 6.58 (s, 1H, Ar–H), 6.64–6.67 (m, 2H, Ar–H), 6.69–6.71 (m, 2H, Ar–H),6.87 (s, 1H, =CH) ppm. ^13^C NMR (DMSO-*d*_6_): *δ* 14.40, 22.43, 26.47, 26.58, 28.69, 31.69, 47.32, 116.70, 116.87, 120.57, 122.73, 123.93, 124.39, 127.68, 127.77, 128.47, 129.25 (2C), 129.80 (2C), 129.90 (2C), 131.11, 132.39, 135.80, 143.54, 147.51, 167.44, 193.93 ppm. Mass analysis (*m*/*z*, %): 516 (M^+^, 14.96), 407 (36.51), 366 (34.28), 335 (100.00), 264 (47.92), 226 (41.87), 145 (41.82). Analysis for C_29_H_28_N_2_OS_3_ (516.74): calculated: C, 67.41; H, 5.46; N, 5.42%. Found: C, 67.54; H, 5.52; N, 5.37%.

#### 2.1.8. 5-((10-Heptyl-10*H*-phenothiazin-3-yl) methylene) pyrimidine-2,4,6(1*H*,3*H*,5*H*)-trione (SR6)

Reddish brown solid (74% yield); m.p. > 300 °C. IR (KBr) *ν*_max_ cm^−1^: 2924–2850 (C–H aliphatic), 1719 (C=O) cm^−1^. ^1^H NMR (DMSO-*d*_6_): *δ* 0.81–0.83 (m, 3H, CH_3_), 1.23–1.36 (m, 8H, CH_2_), 1.62–1.73 (m, 2H, CH_2_), 3.78–3.88 (m, 2H, CH_2_), 5.83 (s, 1H, =CH), 6.71 (s, 1H, Ar–H), 6.84 (s, 2H, Ar–H), 6.89 (t, *J* = 7.20 Hz, 1H, Ar–H), 6.96 (d, *J* = 8.40 Hz, 1H, Ar–H), 7.10 (d, *J* = 7.20 Hz, 1H, Ar–H), 7.17 (t, *J* = 7.60 Hz, 1H, Ar–H), 10.08 (s, 2H, N–H). ^13^C NMR (DMSO-*d*_6_): *δ* 14.40, 21.63, 22.45, 26.70, 28.78, 31.71, 46.80, 91.25, 115.46, 115.82, 122.39, 122.60, 123.99, 125.60, 126.45, 127.49, 127.86, 139.63, 142.03, 145.61, 151.13 (2C), 172.59 (2C) ppm. Mass analysis (*m*/*z*, %): 435 (M^+^, 30.70), 430 (100.00), 376 (76.30), 368 (57.06), 357 (55.41), 351 (62.58), 331 (66.16), 294 (57.18), 280 (60.32), 178 (97.02), 174 (80.85), 133 (54.97), 115 (70.95), 87 (75.61), 86 (87.65). Analysis for C_24_H_25_N_3_O_3_S (435.54): C, 66.19; H, 5.79; N, 9.65%. Found: C, 66.33; H, 5.85; N, 9.74%.

## 3. Results and discussion

### 3.1. Chemistry

**Scheme 1:**
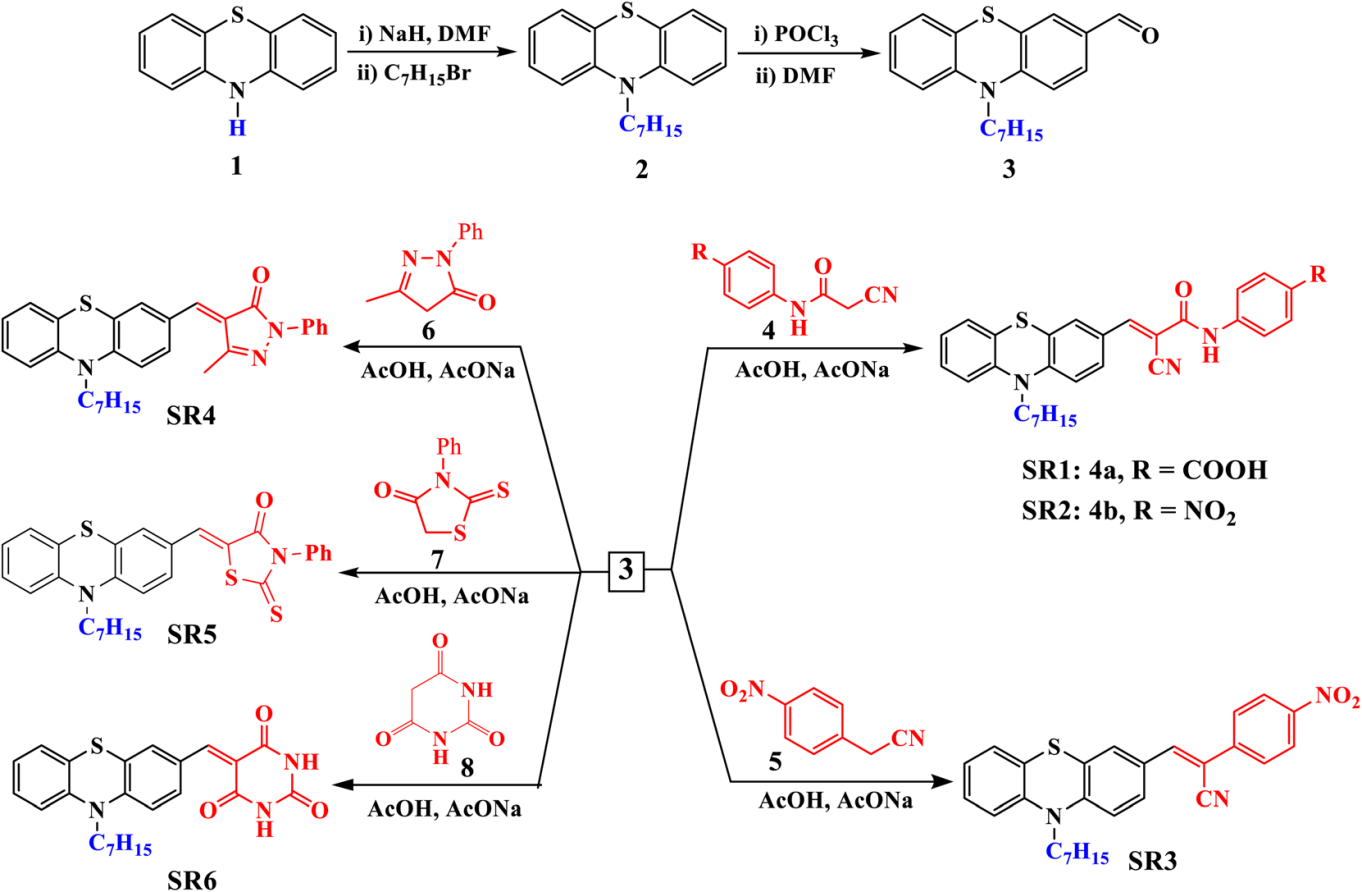
Synthesis of compound 3 and sensitizers SR1–6.

The synthetic routes of the new phenothiazine based organic sensitizers (SR1–6) are depicted in Scheme 1. First, the alkylation reaction between 10*H* phenothiazine (1) using heptyl bromide with NaH in presence of DMF afforded 10-heptyl-10*H*-phenothiazine (2), which undergoes Vilsmeier formylation using POCl_3_ in DMF to yield 10-heptyl-10*H*-phenothiazine-3-carbaldehy (3) with a good overall yield.

1-Cyanoacetyl-3,5-dimethylpyrazole has been utilized as an effective reagent for cyanoacetylation of different primary aromatic amines such as 4-nitroaniline and 4-aminobenzoic acid to furnish the conforming cyanoacetanilide 4a–b as previously described in the literature.^13^ Next, the Knoevenagel reaction between cyanoacetanilides 1a–b and 10-heptyl-10*H*-phenothiazine-3-carbaldehyde (3) in acetic acid in presence of sodium acetate to afford cyanoacetanilide sensitizers SR1–2 (Scheme 1). The other four new organic sensitizers SR3–6 were obtained by Knoevenagel condensation wherein the 10-heptyl-10*H*-phenothiazine-3-carbaldehyde (3) was condensed with different active methylene compounds such as 2-(4-nitrophenyl) acetonitrile (5), 5-methyl-2-phenyl-2,4-dihydro-3*H*-pyrazol-3-one (6), 3-phenyl-2-thioxothiazolidin-4-one (7) and pyrimidine-2,4,6(1*H*,3*H*,5*H*)-trione (8) as shown in Scheme 1.

### 3.2. UV-vis absorption and electrochemical properties

**Fig. 2:**
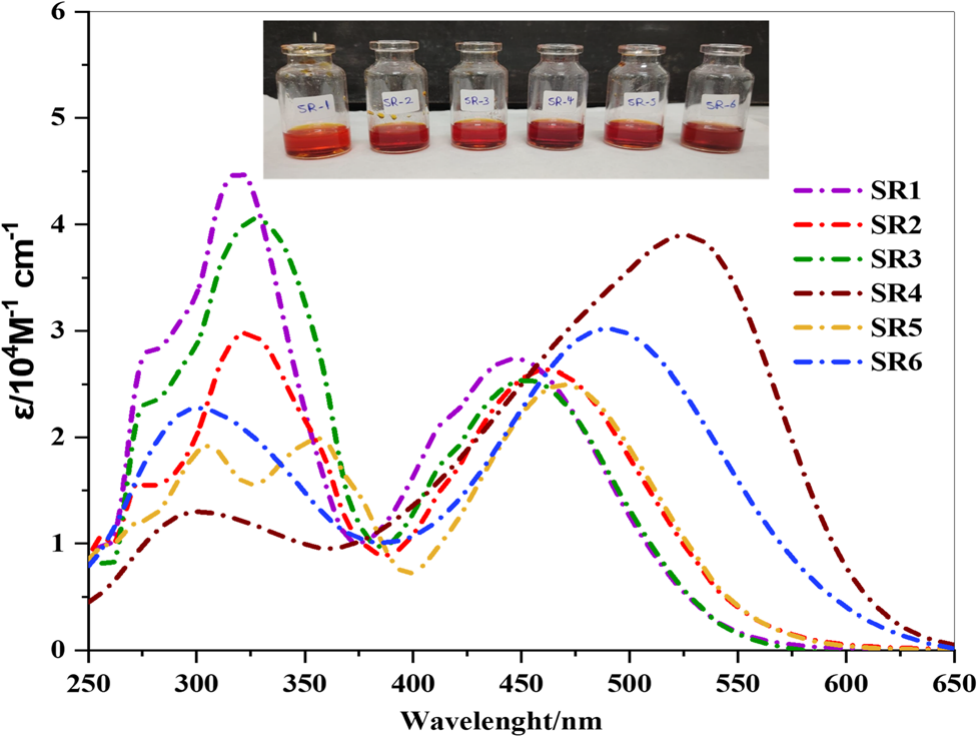
UV-vis absorption spectra of PTZ sensitizers SR1–6 measured in DMF.

The UV-vis absorption spectra of the synthesized phenothiazine sensitizers SR1–6 were analyzed as presented in Table 1. The absorption spectra of SR1–6 have been recorded in DMF solution and are shown in Fig. 2.

**Table 1:** Absorption for phenothiazine sensitizers SR1–6

Sensitizer	*λ*_max_/nm	*ε*/10^4^ M^−1^ cm^−1^	*λ*_onset_/nm	Experimental *E*_0−0_ (eV)
SR1	319, 447	4.48, 2.75	544	2.27
SR2	322, 460	2.98, 2.62	563	2.20
SR3	327, 452	4.06, 2.52	549	2.25
SR4	300, 526	1.30, 3.91	617	2.00
SR5	357, 470	1.98, 2.49	566	2.19
SR6	300, 490	2.28, 3.03	608	2.03

The dyes SR1–6 exhibited two distinct absorption regions, one at shorter wavelengths (250–400 nm) and another at longer wavelengths (420–600 nm). The shorter-wavelength bands were attributed to π–π* electronic transitions, indicative of the conjugated nature of the systems. In contrast, the absorption features in the visible range (420–600 nm) were ascribed to intramolecular charge transfer (ICT) processes between the phenothiazine donor and various acceptor groups, including CN, CO, COOH, and NO_2_.^14^ Specifically, ICT transitions were observed in the 2-cyanacetamide derivatives (CN and NO_2_) of the 2-(4-nitrophenyl) acrylonitrile unit for SR3, the (CO) group within the pyrazol-3-one ring for SR4, the (CN and CS) groups in the thiazolidin-4-one ring for SR5, and the (3 CO) groups in the barbituric ring. The incorporation of a π-bridge was found to enhance light absorption in the visible region, effectively reducing energy gap (*E*_0−0_), as calculated from the onset of the UV-visible absorption spectrum.^14^ Those values followed the order of SR4 < SR6 < SR5 < SR2 < SR3 < SR1. The molar extinction coefficients (*ε*) of the ICT bands of SR1–6 are (2.75, 2.62, 2.52, 3.91, 2.49, and 3.03 × 10^4^ mol^−1^ cm^−1^, respectively). The values of the molar extinction coefficient (*ε*) are significantly greater than those of the Ru dyes N719 (*ε* = 1.08 ×10^4^ M^−1^ cm^1^), indicating a strong capacity for light-harvesting.^15^ Increasing the difference in electronic density between the donating and withdrawing electrons induces a bathochromic shift in the internal charge transfer (ICT) band.^16^ A strong electron-accepting group, on the other hand, should improve the dye's push–pull character and facilitate charge separation within the molecule.^17^ Also, sensitizers, SR4, and SR6 showed significantly greater, red–shifted profiles than SR5. This shift can be attributed to the extended conjugation length within the molecule.^18^ Furthermore, SR4 absorbs at the highest wavelength *λ*_max_ equal to 526 nm at a high value of *ε* at 3.91 × 10^−4^ M^−1^ cm^−1^ which can be explained by the reduced electron delocalization energy of the electron-acceptors and by the presence of five-atom structures (methyl pyrazole), which are smaller in size than the six-member ring.^19^

**Fig. 3:**
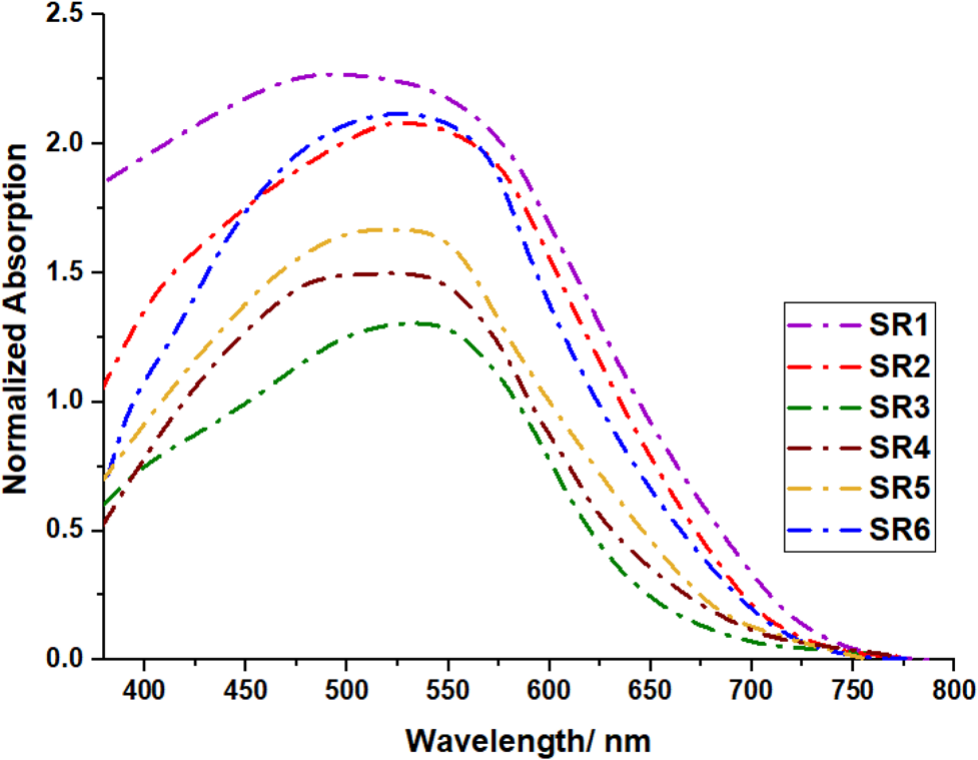
Absorption spectra of SR1–6 adsorbed on nonporous TiO_2_.

When absorbed onto nonporous TiO_2_, the absorption spectra of dyes SR1–6 undergo significant alterations compared to their solution-phase counterparts, illuminating the fundamental interactions between the dyes and the semiconductor surface (Fig. 3). All compounds exhibit pronounced spectral broadening and bathochromic shifts; phenomena attributed to several fundamental photophysical processes. The strong electronic coupling between the carboxylate anchor groups and the TiO_2_ surface leads to efficient orbital mixing, as previously demonstrated by Chen *et al.*^20^ and further supported by recent spectroscopic studies.^21^ This coupling is particularly evident in SR1 and SR6, where the direct connection between the donor and anchor groups facilitates strong electronic interaction with the semiconductor surface. The observed spectral broadening is enhanced by the formation of *J*-aggregates on the TiO_2_ surface, a phenomenon well-documented for similar donor-π-acceptor systems.^22,23^ The non-planar butterfly structure of the phenothiazine core plays a crucial role in determining the electronic properties of these sensitize.^24,25^ In solution, the absorption spectra of SR1–SR6 exhibit distinct variations, which can be attributed to intramolecular charge transfer (ICT) transitions, influenced by the nature of donor–acceptor interactions and solvent effects. However, when adsorbed on TiO_2_, most of the sensitizers display similar spectral features, except for SR4, which remains almost unshifted. Sensitizers SR1, SR2, SR3, SR5, and SR6 possess highly conjugated acceptor groups (COOH, CN, CS, or barbituric rings) that facilitate stronger charge transfer interactions upon adsorption. SR4, however, contains a pyrazolone ring, which may alter its adsorption geometry, reducing direct electronic overlap with TiO_2_ and thus minimizing shifts in its absorption spectrum. The observed spectral similarities upon TiO_2_ adsorption, aside from SR4, can be attributed to strong electronic coupling, *J*-aggregation effects, and charge transfer interactions, leading to spectral broadening and red shifts. The unique behavior of SR4 suggests that its molecular structure restricts significant interaction with TiO_2_, preserving its solution-phase absorption properties.

**Fig. 4:**
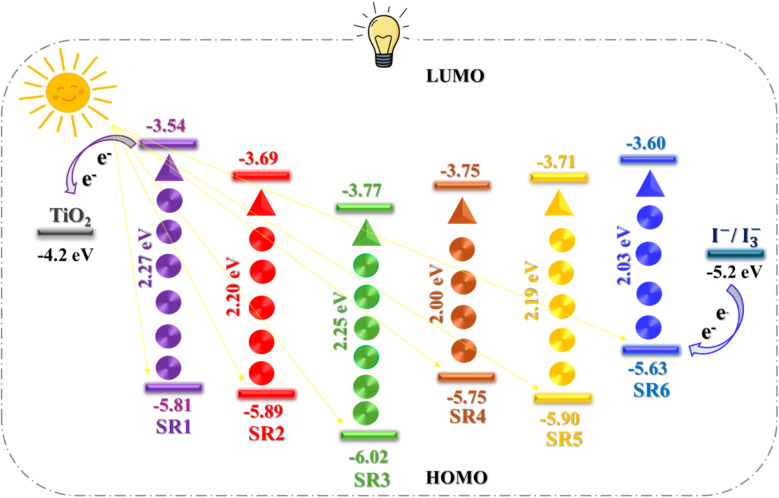
Energy level diagram for SR1–6 sensitizers.

To assess the potential for dye regeneration and electron injection, cyclic voltammetry (CV) experiments were performed in THF with TBAPF_6_ as the supporting electrolyte, as illustrated in Fig. (S26†). The results, summarized in Table (S1†), reveal that the energy levels of the dyes are well-aligned for efficient operation in dye-sensitized solar cells (DSSCs). The ground-state oxidation potentials (GSOP) of the compounds, spanning from −5.75 to −6.02 eV, are significantly more negative than the *I*^−^/*I*_3_^−^ redox couple (−5.2 eV), ensuring an adequate driving force for effective dye regeneration.^26,27^ The excited state oxidation potentials (ESOP), calculated from GSOP levels and *E*_0−0_ values, range from −3.54 to −3.77 eV, positioning them favorably above the TiO_2_ conduction band (−4.2 eV). This energy level alignment provides adequate driving force for electron injection, as demonstrated by Zhang and coworkers in related systems as shown in (Fig. 4).^28,29^ The experimental values show excellent agreement with theoretical calculations, validating our molecular design strategy and energy level engineering approach.^30^

### 3.3. Theoretical calculations

#### 3.3.1. Molecular modeling

**Fig. 5:**
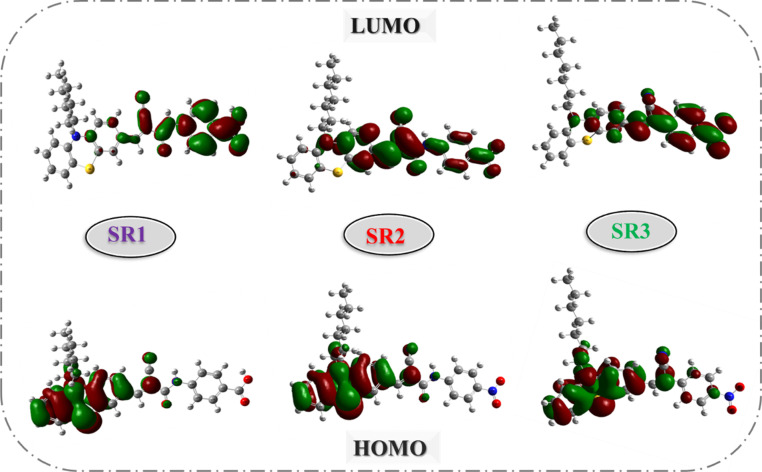
HOMOs and LUMOs geometry for SR1–2.

The SR1–6 sensitizers were subjected to density-functional theory (DFT) calculations utilizing the B3LYP with the d-polarized 6-311G basis that is implemented in the Gaussian09 program.^31^ As shown in Fig. S27,† the optimized structure for compounds SR1–6, and elucidate the relationship between the six SR1–6 sensitizer dyes' geometric structure and Fig. 5 and6 the electrical distribution of their HOMO and LUMO levels in additional detail. For the SR1 sensitizer, the HOMO electron density was mostly localized on the donor parts (phenothiazine ring), while electronic densities of LUMO were mostly localized on CN, CO, and COOH segments. But in the case of SR2, the electron density of LUMO extended towards the acceptor moieties (CN, CO, and NO_2_). Also, the HOMO of SR3 was mostly concentrated in the donor parts (phenothiazine ring), while the 4-nitroacetonitrile acceptor moiety (CN and NO_2_) was where the majority of the LUMO electron density was located. For SR5 introduction thiazolidine-4-one ring facilitates the transfer of electrons from the phenothiazine moiety's donor side to its acceptor side that is centered on the (C=S and carbonyl) segments. In the case of SR6, the barbituric acid anchoring part possesses the LUMO electron density.

**Fig. 6:**
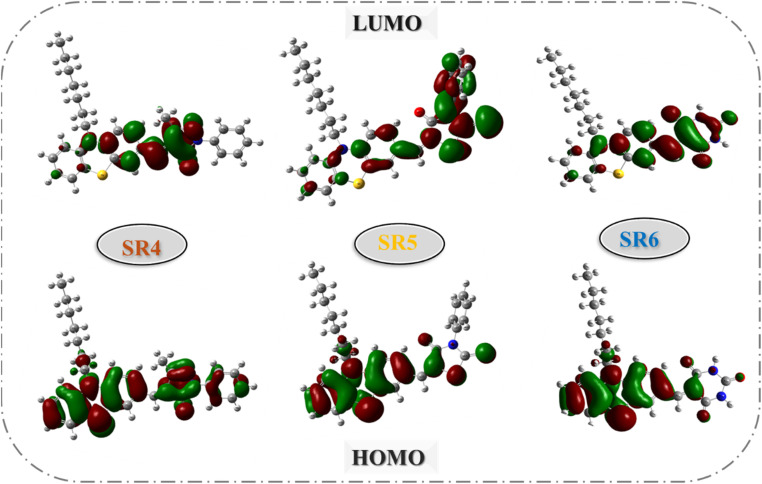
HOMOs and LUMOs geometry for SR3–6.

#### 3.3.2. Molecular electrostatic potential (MEP)

**Fig. 7:**
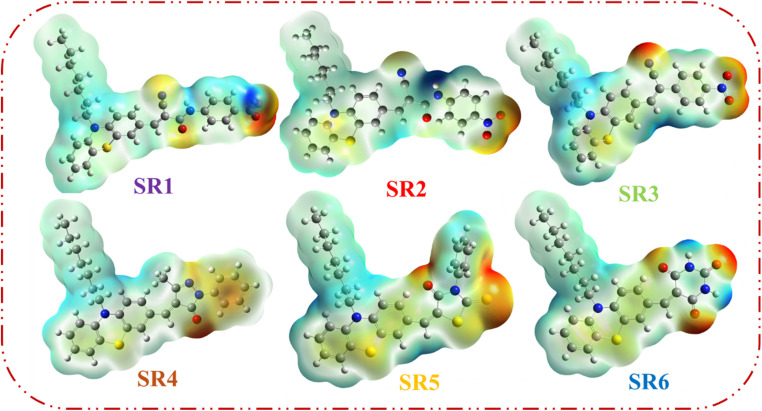
Molecular electronic potential diagram (MEP) of sensitizers SR1–6.

From the cube file produced by Gaussian computations, molecular electrostatic potentials (MEPs) can be extracted as a useful tool for identifying organic molecule internal charge transfer (ICT) properties, in this case between the HOMO and LUMO levels of phenothiazine in SR1–6 dyes.^32^Fig. 7 shows the results of analyzing the HOMO–LUMO levels and MEPS of all SR1–6 sensitizers to determine the impact of donor–acceptor groups. Electrophilic activity is found in the red parts of the MEP, which are related to electron-rich regions, while nucleophilic activity is found in the blue parts, which are associated with electron-deficient portions of sensitizers SR1–6. Electrostatic potential increased in the following order: red, orange, yellow, green, and blue. For the SR1 sensitizer, which incorporates a cyanoacetamide moiety, the negative (red) charge is primarily localized on the cyano group and the carbonyl group connected to the COOH moiety. In SR2, the negative (red) low potential is predominantly concentrated in the region of the anchoring group, specifically within the cyano, carbonyl, and nitro groups. Similarly, in SR3, the negative (red) low potential is mainly observed around the anchoring group, with a particular focus on the cyano (CN) and nitro (NO_2_) groups. In contrast, for SR4, the pyrazole ring's carbonyl (CO) group is identified as the main site of the negative charge. The carbonyl group on the thiazolidine-4-one ring and the C=S group are the specific locations where the red color is concentrated for the SR5 sensitizer. Finally, SR6, the negative (red) low potentials concentrated on the carbonyl groups of the barbituric ring. The positive region (blue) of the MEP map is found across the donor heptyl phenothiazine region, indicating that they are favorable sites for nucleophilic attack.

### 3.4. Photovoltaic performance for sensitizers SR1–6

**Fig. 8:**
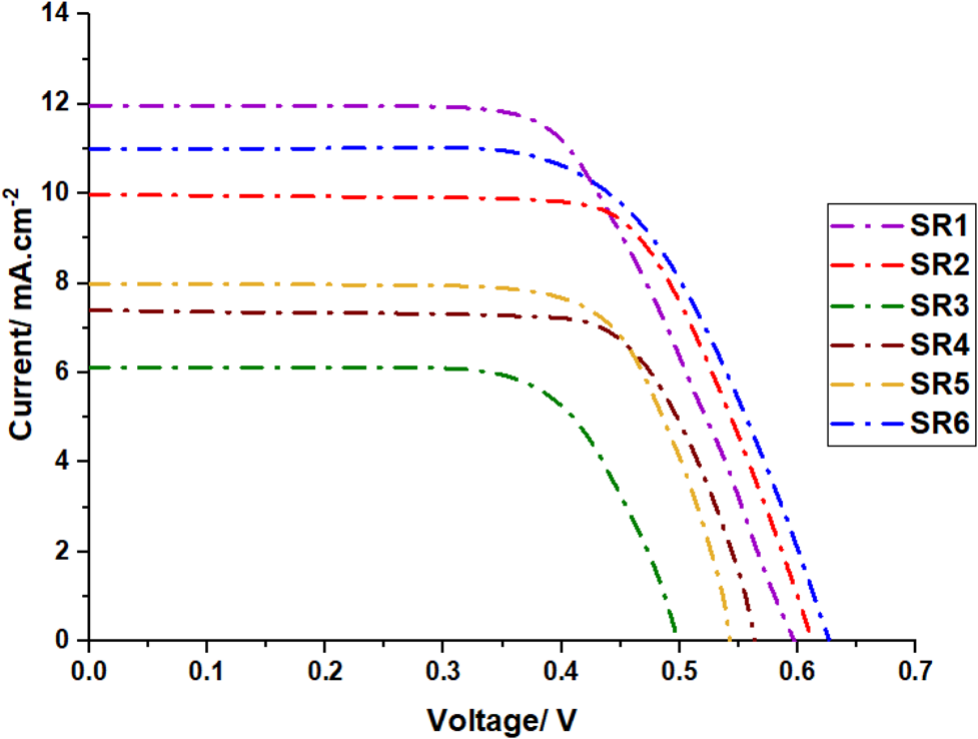
*I*–*V* characteristics of solar devices based on SR1–6.

The photovoltaic properties of phenothiazine sensitizers SR1–6 were systematically evaluated under standard conditions using AM 1.5 G. The current density–voltage (*J*–*V*) characteristics are illustrated in Fig. 8, with the detailed performance parameters summarized in Table 2. Dye loading experiments are commonly used to better realize the effect of several anchors on dye performance. Considering this, a DMF/H_2_O (1 : 1) combination containing 0.1 M NaOH was used to desorb dye from the TiO_2_ surface to quantify the total quantity of dye adsorbed on the TiO_2._

**Table 2:** Photovoltaic parameters of compounds SR1–6. Significance values are in bold

Sensitizers	*J*_SC_ a (*J*_SC_^b^) (mA cm^−2^)	*V*_OC_ a (*V*_OC_^b^)/mV	FF^a^ (FF^b^)/%	PCE^a^ (PCE^b^)/%	Concentration of the dye/10^−5^ mol cm^−2^
SR1	**11.96** (**11.88** ± **0.168**)	**0.597** (**0.594** ± **0.005**)	**59.20** (**59.43** ± **1.69**)	**4.22** (**4.21** ± **0.031**)	**0.93**
SR2	**9.95** (**9.92** ± **0.046**)	**0.611** (**0.602** ± **0.017**)	**57.30** (**58.033** ± **1.97**)	**3.48 (3.47**±**0.052**)	**0.80**
SR3	**6.08** (**6.02** ± **0.105**)	**0.499** (**0.496** ± **0.005**)	**60.40** (**61.053** ± **1.18**)	**1.83** (**1.816** ± **0.031**)	0.67
SR4	**7.98** (**7.94** ± **0.056**)	**0.563** (**0.562** ± **0.005**)	**58.9** (**58.58** ± **0.955**)	**2.64** (**2.623** ± **0.0318**)	**0.55**
SR5	**7.40** (**7.37**±**0.052**)	**0.543** (**0.540** ± **0.005**)	**62.5** (**63.2** ± **1.153**)	**2.51** (**2.513** ± **0.046**)	**0.50**
SR6	**10.99** (**10.96** ± **0.055**)	**0.626** (**0.623** ± **0.006**)	**56.4** (**56.05** ± **0.551**)	**3.88** (**3.88** ± **0.041**)	**0.89**

a The best device parameters (listed in the manuscript).

b The average device parameters (obtained from three devices).

The photovoltaic parameters of single-dye devices reveal complex structure–performance relationships that provide valuable insights into molecular design principles. SR1 achieves the highest efficiency (4.22%) among the series, characterized by a short-circuit current density (*J*_sc_) of 11.96 mA cm^−2^, open-circuit voltage (*V*_oc_) of 0.597 V, and fill factor (FF) of 59.2%. This superior performance stems from optimal energy level alignment and efficient charge injection dynamics.^33,34^ The direct connection between the donor and anchor groups in SR1 facilitates rapid electron injection into the TiO_2_ conduction band while maintaining sufficient driving force for dye regeneration.^35^SR3 exhibits the lowest efficiency (1.83%) with significantly reduced photovoltaic parameters (*J*_sc_ = 6.08 mA cm^−2^, *V*_oc_ = 0.499 V, FF = 60.4%). The poor performance is attributed to the weak anchoring ability of the nitroacetonitrile acceptor moiety, which limits effective electron injection and interfacial interaction with TiO_2_.^36^ The reduced *V*_oc_ indicates increased charge recombination rates, likely due to the formation of surface trap states at the TiO_2_/dye interface.^37^ Among the single-dye devices, SR1 exhibited the highest efficiency (4.22%), followed by SR6 (3.88%), due to their optimal donor-π-acceptor interactions, favorable energy level alignment, and strong charge injection capabilities and the strength of the acceptors moieties, while SR3 performed the lowest (1.83%), likely due to its weak nitroacetonitrile acceptor, which hinders effective electron injection and increases recombination losses and the lower dye loading of the dye. Sensitizers SR2 (3.48%), SR4 (2.64%), and SR5 (2.51%) showed moderate efficiencies, demonstrating that molecular structures with better anchoring ability and charge transfer dynamics tend to enhance performance, the photovoltaic performance agree with the absorbance of TiO_2_.

**Fig. 9:**
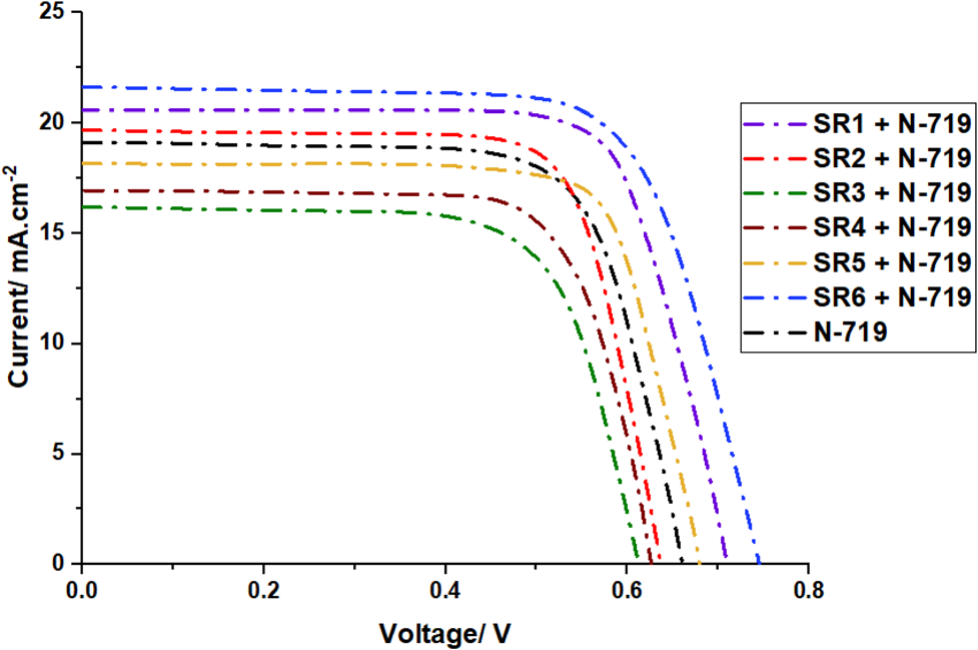
*I*–*V* characteristics of solar devices sensitized with N-719 and SR1–6.

The cocktail co-sensitization strategy with N719 demonstrates remarkable enhancement in device performance through complementary absorption and reduced aggregation effects. The SR6 + N719 system achieves an unprecedented efficiency of 9.77%, characterized by significantly improved photovoltaic parameters (*J*_sc_ = 21.63 mA cm^−2^, *V*_oc_ = 0.743 V, FF = 60.8%). This exceptional performance represents a 33.3% improvement over standard N719 devices (*η* = 7.33%) and can be attributed to several synergistic effects^38,39^ as shown in Table 3 and Fig. 9.

**Table 3:** Photovoltaic parameters of the SR1–6 + N-719 and N-719 only. Significance values are in bold

Cell device	*J*_SC_ a (*J*_SC_^b^) (mA cm^−2^)	*V*_OC_ a (*V*_OC_^b^)/mV	FF^a^ (FF^b^)/%	PCE^a^(PCE^b^)/%	Concentration of the dye/10^−5^ mol cm^−2^
SR1 + N-719	**20.53** (**20.55** ± **0.066**)	**0.709** (**0.714** ± **0.012**)	**58.6** (**58.28** ± **0.649**)	**8.52** (**8.56** ± **0.193**)	**2.52**
SR2 + N-719	**19.70** (**19.70** ± **0.129**)	**0.637** (**0.637** ± **0.016**)	**56.9** (**56.89** ± **0.188**)	**7.14** (**7.146** ± **0.147**)	**1.65**
SR3 + N-719	**16.19** (**16.27** ± **0.142**)	**0.609** (**0.617** ± **0.026**)	**57.90** (**57.96** ± **0.284**)	**5.70** (**5.69** ± **0.115**)	**1.44**
SR4 + N-719	**16.93** (**16.89** ± **0.095**)	**0.626** (**0.635** ± **0.019**)	**58.70** (**58.69** ± **0.198**)	**6.22** (**6.20** ± **0.036**)	**1.65**
SR5 + N-719	**18.23** (**18.18** ± **0.118**)	**0.680** (**0.685** ± **0.011**)	**60.10** (**60.27** ± **0.402**)	**7.45** (**7.53** ± **0.228**)	**1.77**
SR6 + N-719	**21.63** (**21.56** ± **0.474**)	**0.743** (**0.744** ± **0.006**)	**60.80** (**60.82** ± **0.717**)	**9.77** (**9.70** ± **0.126**)	**2.54**
N-719	**19.07** (**19.11** ± **0.220**)	**0.660** (**0.662** ± **0.006**)	**58.30** (**58.163** ± **0.344**)	**7.33** (**7.47** ± **0.374**)	**1.87**

a The best device parameters (listed in the manuscript).

b The average device parameters (obtained from three devices).

The variation in cocktail co-sensitization efficiency among SR dyes can be directly linked to their molecular structures and resulting electronic properties. SR6 exhibits optimal GSOP (−5.75 eV) and ESOP (−3.75 eV) levels, positioning it ideally relative to the TiO_2_ conduction band (−4.2 eV) and the electrolyte redox potential (−5.2 eV).^3,4^ This energy level alignment facilitates efficient electron injection into TiO_2_ while maintaining robust dye regeneration capabilities. The broad absorption spectrum of SR6 on TiO_2_, complementing N719's absorption profile, enables enhanced light harvesting across the visible spectrum.^40,41^ As shown in Fig. 9, SR1 and SR5, showing similarly impressive co-sensitization performance (*η* = 8.52% and 7.45% respectively), demonstrate comparable electronic characteristics. Their GSOP values (−5.81 eV and −5.90 eV) and ESOP levels (−3.54 eV and −3.71 eV) create favorable energy cascades for electron injection and dye regeneration.^42^ The broadening and red-shifting of absorption spectra observed when SR dyes are adsorbed on TiO_2_ indicates strong electronic coupling between the dye molecules and the semiconductor surface. This phenomenon is particularly pronounced in SR6, SR1, and SR5, where the extended π-conjugation systems facilitate better electronic communication with both TiO_2_ and N719.^43,44^ The lower performance of SR3 + N719 (*η* = 5.70%) correlates with its less favorable GSOP (−6.02 eV) and ESOP (−3.77 eV) levels, resulting in suboptimal electron injection dynamics.^10,11^ This is reflected in its lower *R*_ct_ value (41.52 Ω) as shown in the EIS studies and reduced photovoltaic parameters (*J*_sc_ = 16.19 mA cm^−2^, *V*_oc_ = 0.609 V).^45^ The underperformance of SR2–4 in cocktail co-sensitization can be attributed to less favorable molecular arrangements and electronic coupling, resulting in increased charge recombination (lower *R*_ct_ values) and reduced light harvesting efficiency.^46^ Upon co-sensitization with N719, the overall efficiencies significantly improved, with SR6 + N719 achieving the highest performance (9.77%), followed by SR1 + N719 (8.52%) and SR5 + N719 (7.45%), attributed to their complementary light absorption, enhanced charge separation, and reduced recombination, as indicated by their higher charge transfer resistance (*R*_ct_) values from electrochemical impedance spectroscopy (EIS) studies. Conversely, SR3 + N719 exhibited the lowest efficiency (5.70%), reaffirming its poor electron injection dynamics and higher charge recombination rates, while SR2 + N719 (7.14%) and SR4 + N719 (6.22%) displayed moderate enhancements. The photovoltaic trends directly correlate with their molecular structures, where dyes with extended conjugation, stronger anchoring groups, and optimized energy level alignment consistently outperformed others. Additionally, higher *R*_ct_ values in co-sensitized devices (SR6 + N719: 49.98 Ω, SR1 + N719: 48.17 Ω) indicate suppressed electron recombination, leading to improved (*V*_oc_) and overall efficiency. The comprehensive analysis highlights that efficient co-sensitization relies on synergistic energy level alignment, complementary spectral absorption, and minimized recombination losses, providing valuable insights for the rational design of next-generation DSSC sensitizers with enhanced light-harvesting and charge transport properties.

**Fig. 10:**
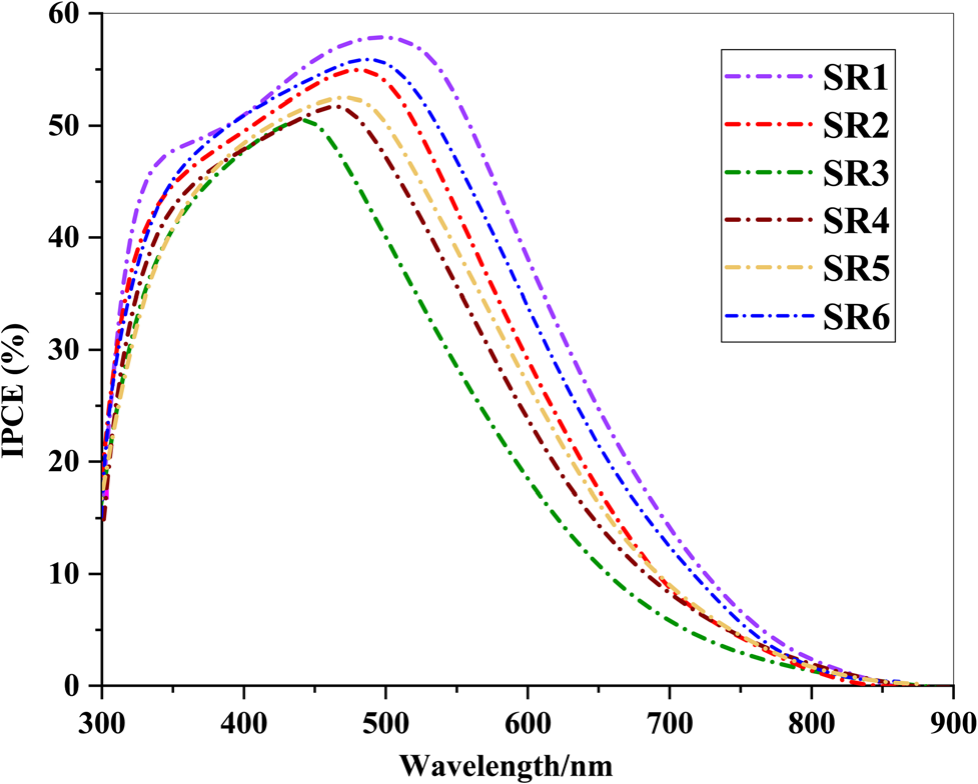
IPCE spectra of phenothiazine-sensitizer SR1–6.

The incident photon-to-current efficiency (IPCE) spectra of the phenothiazine-based sensitizers SR1–6 revealed significant differences in their light-harvesting and electron injection efficiencies as shown in Fig. 10.^46^ The single-dye DSSCs exhibited broad photoresponse in the 300–600 nm range, with IPCE maxima aligning with their UV-vis absorption peaks. Among them, SR1 displayed the highest IPCE values (56% at 500 nm), indicating strong light absorption and efficient charge injection into TiO_2_. In contrast, SR3 exhibited the lowest IPCE (40%), likely due to its weaker electron-withdrawing acceptor, leading to inefficient charge transfer and increased recombination. The IPCE integral areas of DSSCs exhibit an order for dyes of SR1 > SR6 > SR2 > SR5 > SR4 > SR3, which is consistent with the trend of *J*_SC_.

**Fig. 11:**
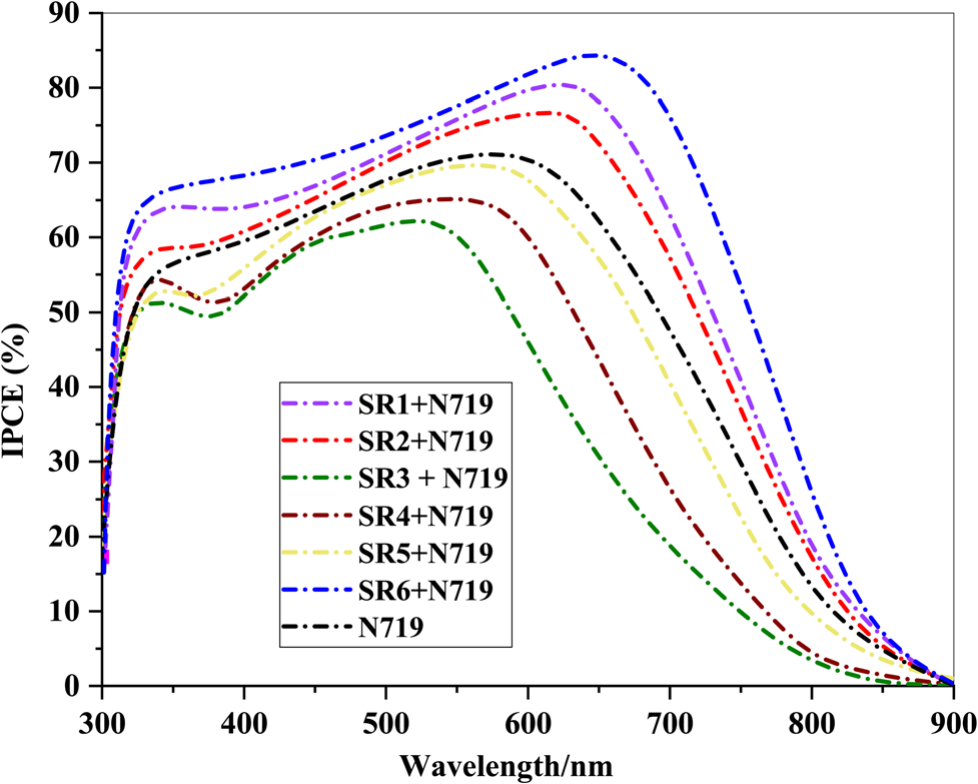
IPCE spectra of phenothiazine-sensitizer SR1–6 + N719 and N719.

Upon co-sensitization with N719, the IPCE spectra showed a substantial increase, particularly in the 450–600 nm range, confirming improved spectral utilization and enhanced photocurrent generation as shown in Fig. 11. Notably, the SR6 + N719 system achieved the highest IPCE (∼85% at 500 nm), reflecting superior light absorption, efficient charge injection, and reduced recombination losses. The enhanced IPCE response in co-sensitized devices is consistent with their higher (*J*_sc_) and (*R*_ct_), as confirmed by (EIS), which indicates suppressed recombination and improved electron transport. These results emphasize the importance of co-sensitization in broadening the absorption spectrum, enhancing charge separation, and boosting overall DSSC efficiency.

### 3.5. Charge transfer dynamics

**Fig. 12:**
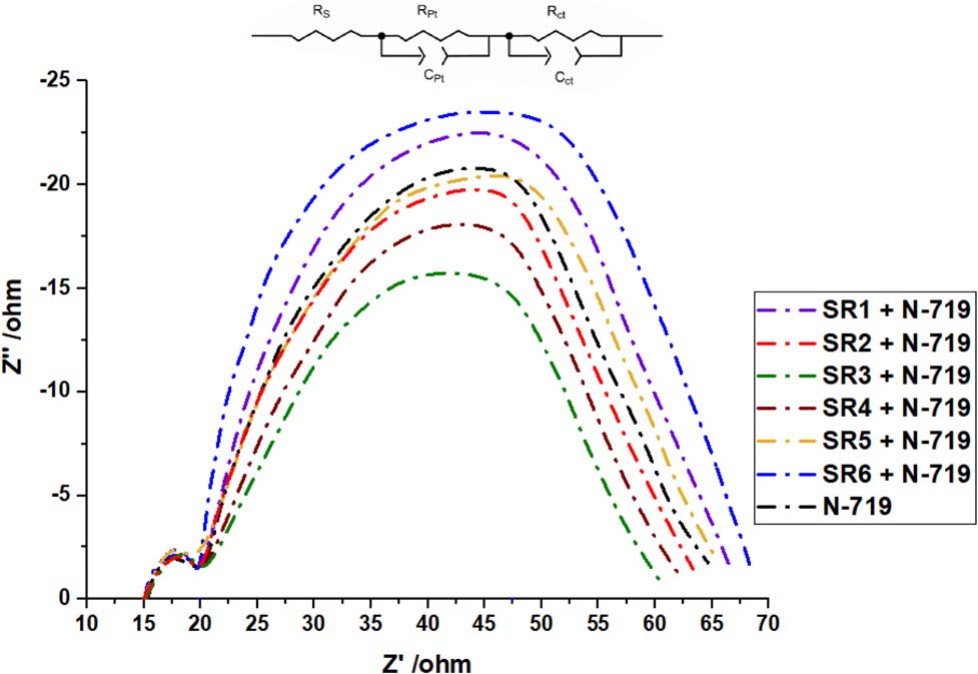
Nyquist plots of SR1–6 + N719-based devices.

To further investigate the mechanisms of charge recombination in DSSC configurations, electrochemical impedance spectroscopy (EIS) was performed under a forward bias of 0.70 V in the absence of illumination.^47–50^ The Nyquist plots, presented in Fig. 12, display two characteristic semicircles. The smaller semicircle observed in the high-frequency region corresponds to the charge-transfer impedance (*R*_Pt_) at the Pt counter electrode, while the larger semicircle in the intermediate-frequency range represents the charge-transfer resistance *R*_ct_ at the TiO_2_/sensitizer/electrolyte interface. An increased *R*_ct_ value indicates reduced electron recombination, which contributes to an enhancement in the open-circuit voltage (*V*_OC_). Additionally, *Rs* represents the series resistance. The values of *R*_Pt_, *R*_ct_, and *R*_s_ were determined by fitting the experimental data to an equivalent circuit model, as shown in the inset of Fig. 11.^51^ All DSSC devices exhibited comparable *R*_s_ and *R*_Pt_ values, reflecting the consistent use of identical FTO substrates and Pt electrodes. However, *R*_ct_ values varied across the devices, following the order: SR6 + N-719 (49.98 Ω) > SR1 + N-719 (48.17 Ω) > SR5 + N-719 (47.22 Ω) > N-719 (46.08 Ω) > SR2 + N-719 (44.56 Ω) > SR4 + N-719 (43.24 Ω) > SR3 + N-719 (41.52 Ω). This trend is consistent with the observed variations in *V*_OC_, as summarized in Table 3. Notably, the co-sensitized devices (SR6, SR1, and SR5) demonstrated higher *R*_ct_ values compared to devices employing N-719 alone, indicating a suppression of electron recombination between injected electrons and the electrolyte. This behavior is likely attributed to the formation of a more compact and organized monolayer of sensitizers, facilitated by enhanced dye adsorption.^52,53^ Also the Nyquist plots for sensitizers SR1–6 presented in Fig. (S28),† it observed that *R*_ct_ values varied across the devices, following the order: SR6 > SR2 > SR1 > SR4 > SR5 > SR3. This trend is consistent with the observed variations in *V*_OC_, as summarized in Table 2.

## 4. Conclusion

This study provides a detailed investigation of phenothiazine-based sensitizers (SR1–6) for dye-sensitized solar cells (DSSCs), focusing on their spectral, electrochemical, and photovoltaic properties in both single-dye and co-sensitization configurations. While co-sensitization is an established approach in DSSCs, our findings offer new insights into how specific molecular structures influence device performance, particularly in enhancing light absorption, charge transfer dynamics, and interface stability. Among the single-dye devices, SR1 demonstrated the highest efficiency (4.22%), followed by SR6 (3.88%), emphasizing the importance of donor–acceptor interactions and energy level alignment. When co-sensitized with N719, SR6 + N719 achieved a notable efficiency of 9.77%, surpassing N719-only devices (7.33%). The electrochemical impedance spectroscopy (EIS), where SR6 + N719 exhibited the highest charge transfer resistance (*R*_ct_ = 49.98 Ω), indicating suppressed electron recombination and enhanced interfacial charge transport.

## Consent to participate

All authors participated directly in the current research work.

## Data availability

The data supporting this article have been included as part of the ESI.†

## Conflicts of interest

Authors declare that they have no conflict of interest.

## Supplementary Material

RA-015-D5RA00694E-s001
