# Supplementary material for: Novel phenothiazine-based sensitizers for high-performance dye-sensitized solar cells: enhanced photovoltaic properties through strategic Co-sensitization with N719
Source: RSC Adv. 2025 Apr 30;15(18):13896–907. doi: 10.1039/d5ra00694e (PMC12041852; doi:10.1039/d5ra00694e)
Supplement: RA-015-D5RA00694E-s001 [file RA-015-D5RA00694E-s001.pdf]

Supplementary Information (SI) for RSC Advances.
This journal is © The Royal Society of Chemistry 2025

Novel Phenothiazine-Based Sensitizers for High-Performance Dye-Sensitized Solar Cells: Enhanced Photovoltaic Properties through Strategic Co-sensitization with N719

Sara H. Yousef[a], Ehab Abdel-Latif[a*], Safa A. Badawy[a], Mohamed R. Elmorsy[a]

[a] *Department of Chemistry, Faculty of Science, Mansoura University, Mansoura 35516, Egypt.*

**Corresponding author: E-mail: ehabattia00@gmx.net*

Supporting Information

1. Experimental general remarks:

Melting points were determined with Gallenkamp melting point apparatus and are uncorrected. The infrared (IR) spectra were recorded on Termo Scientifc Nicolet iS10 FTIR. ^{1}H NMR and ^{13}C NMR spectra were recorded DMSO-d_6 as a solvent using JEOL's spectrometer at 500 MHz or Brucker's spectrometer at 400 MHz using tetramethylsilane (TMS) as internal standard. Chemical shifts are expressed in δ, ppm. ^{1} HNMR data are reported in order: multiplicity (br, broad; s, singlet; d, doublet; t, triplet; dd, doublet of doublet; m, multiplet), approximate coupling constant in Hertz, number of protons and type of protons. The purity of the compounds was checked by ^{1}H NMR and thin layer chromatography (TLC) on silica gel plates using a mixture of dichloromethane and methanol or petroleum ether and ethyl acetate as eluent. UV lamp was used as a visualizing agent. Mass analyses and elemental analyses were recorded on Termo DSQ II spectrometer at Faculty of Science, Al-Azhar University

2. Analytical Measurements

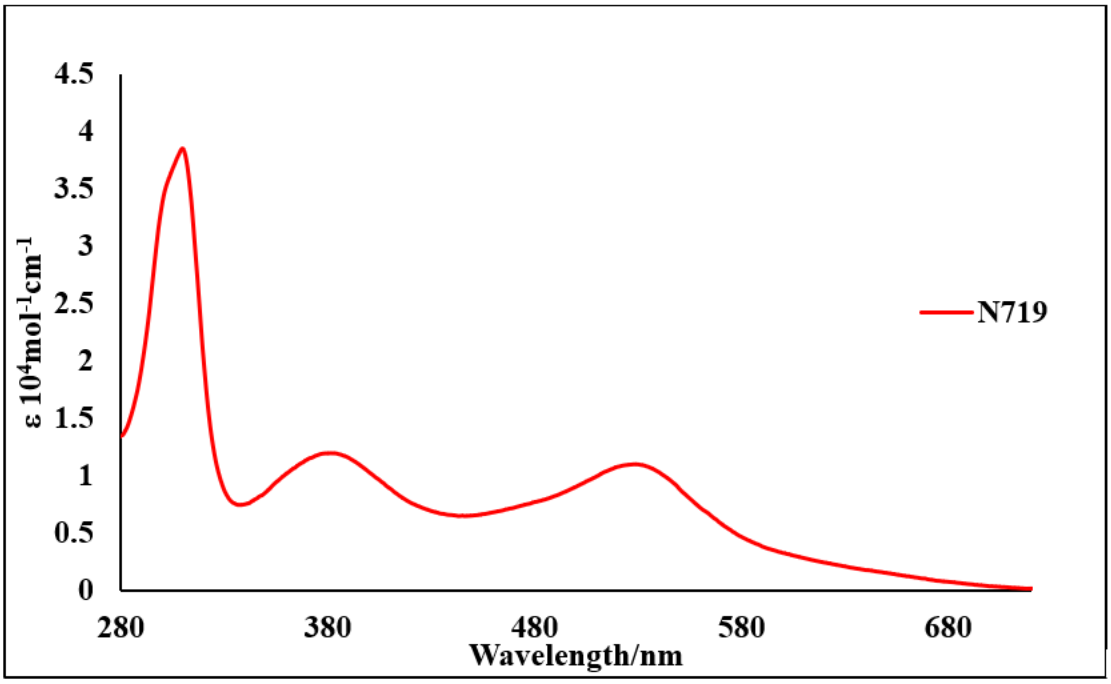

Figure (S1). UV-Vis. absorption of N719.

λ_{max} (nm)	ε ($10^4 M^{-1} cm^{-1}$)	λ_{onset} / nm	E_{0-0} (ev)
307, 384, 537	3.69, 1.18, 1.08	602	1.92

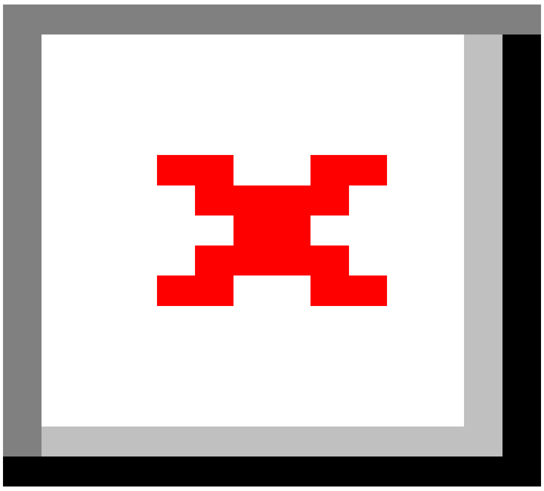

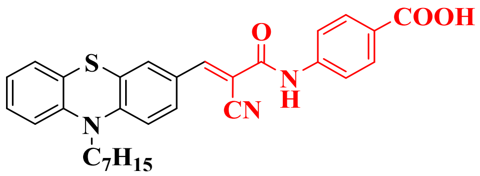

Figure (S2): IR spectrum of sensitizer SR1.

https://drive.google.com/drive/folders/1heYOp66P8AN6PxPWAAC5hDvDgOs1bVDU?usp=sharing

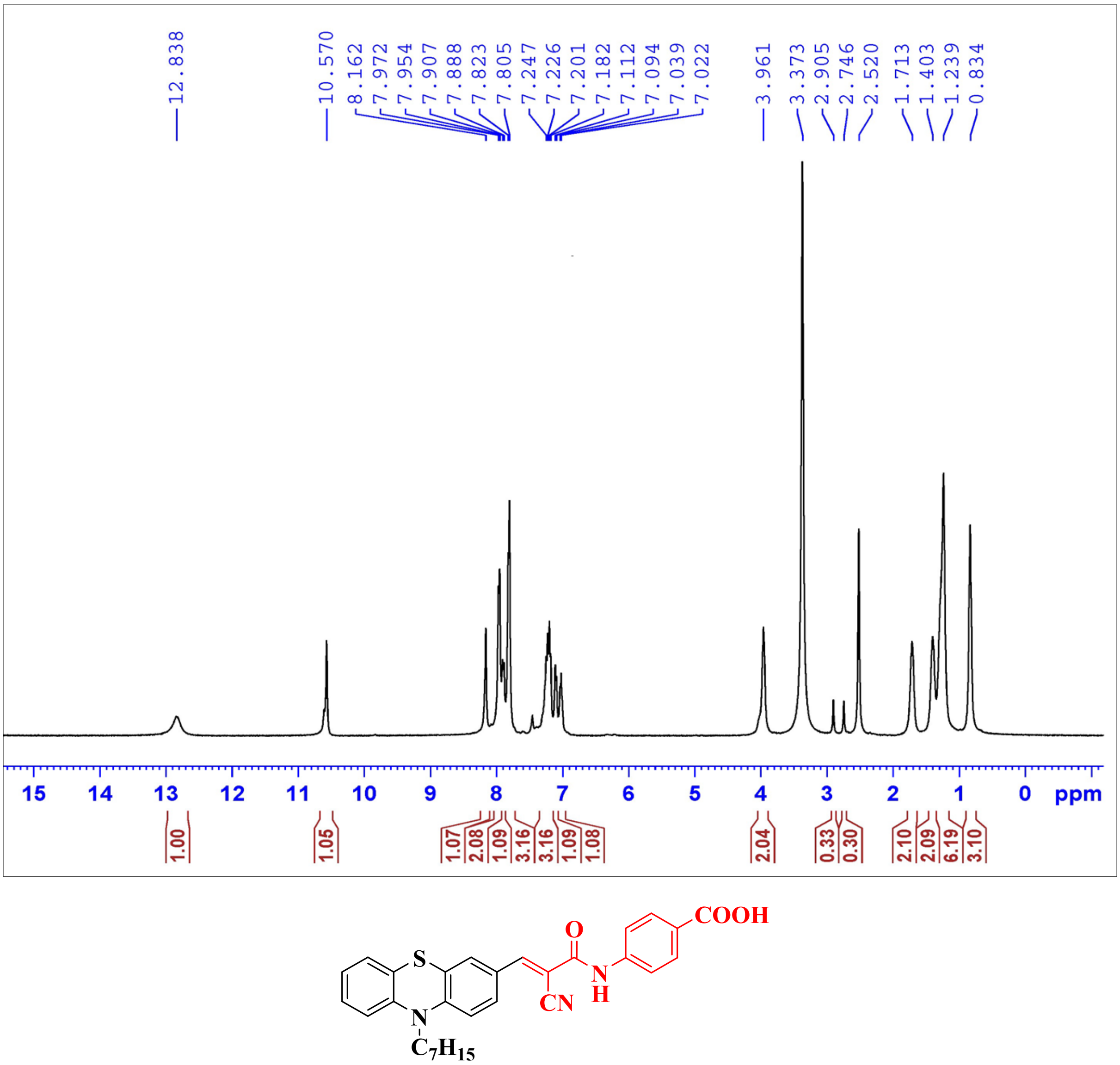

Figure (S3-A): ^{1}H NMR spectrum of sensitizer SR1.

https://drive.google.com/drive/folders/1z0ba20wNbsYZhqHApoAeDTyMbNieSSOD?usp=sharing

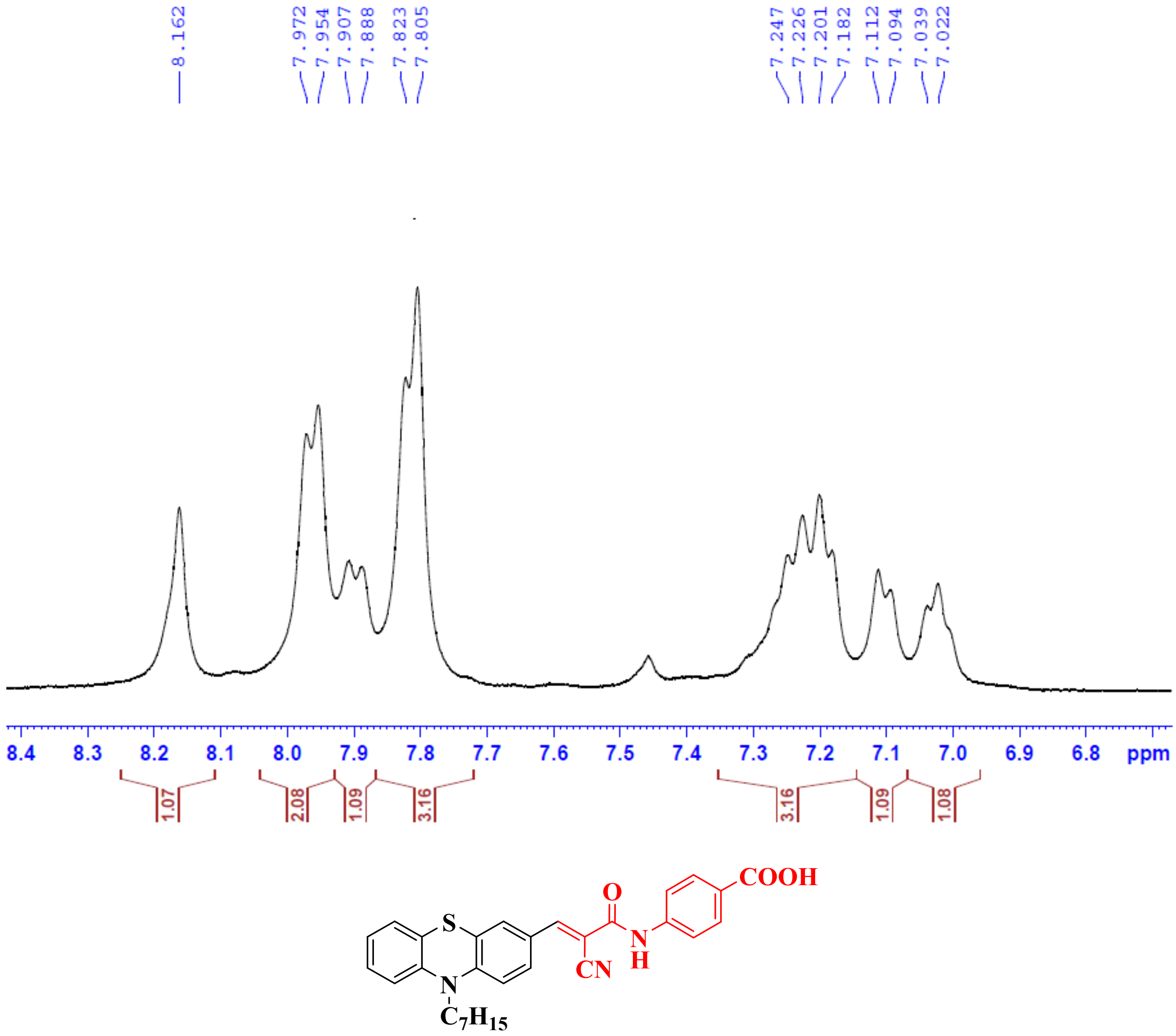

Figure (S3-B): ^{1}H NMR spectrum of sensitizer SR1.

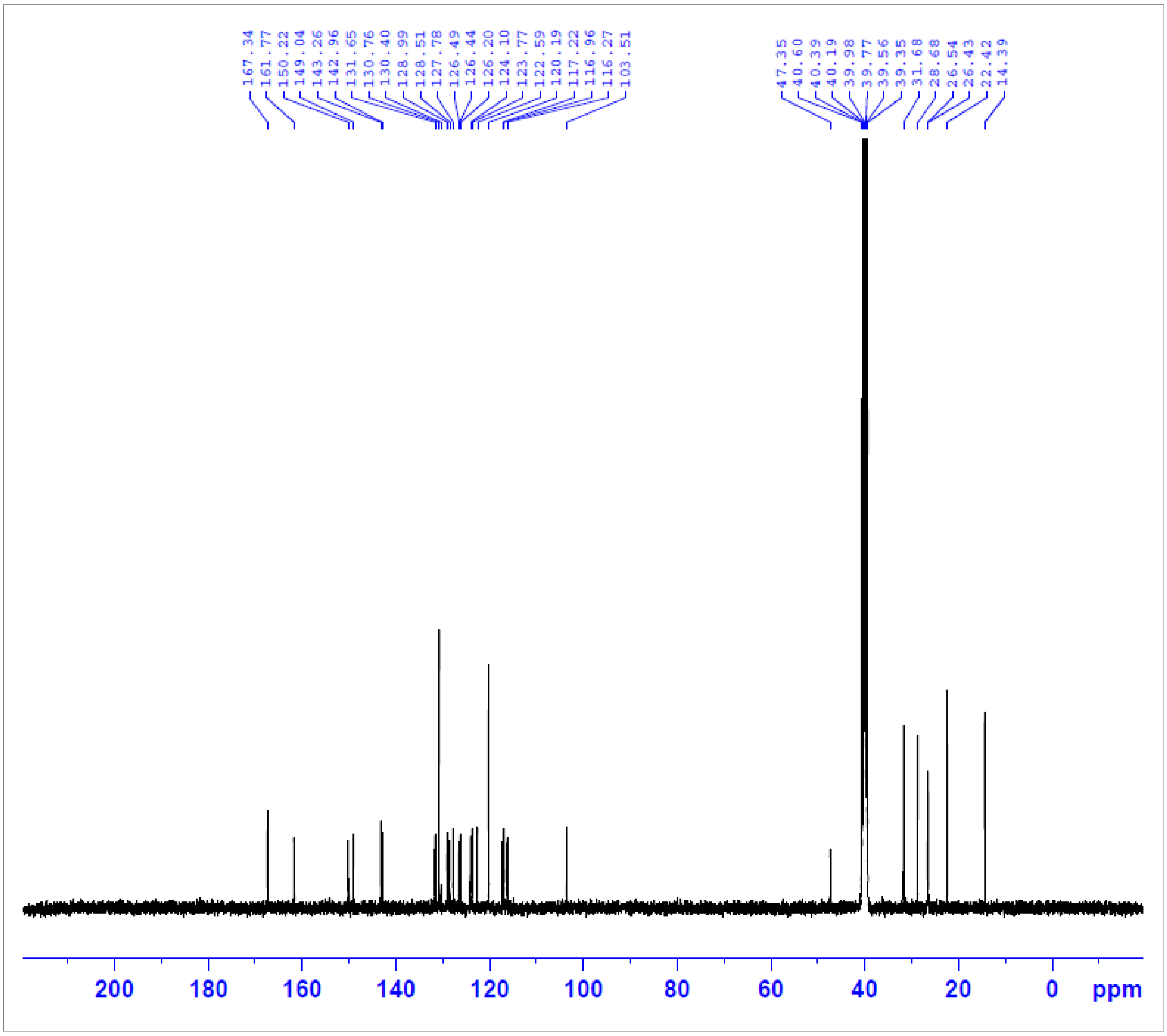

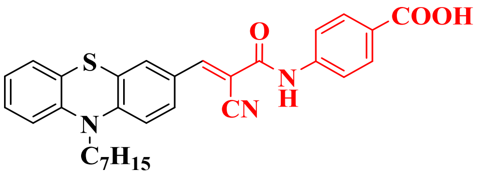

Figure (S4): ^{13}C NMR spectrum of sensitizer SR1.

https://drive.google.com/drive/folders/1IS5uCcnKevWNsuvfwqwbtVS0LV_N0yXc?usp=sharing

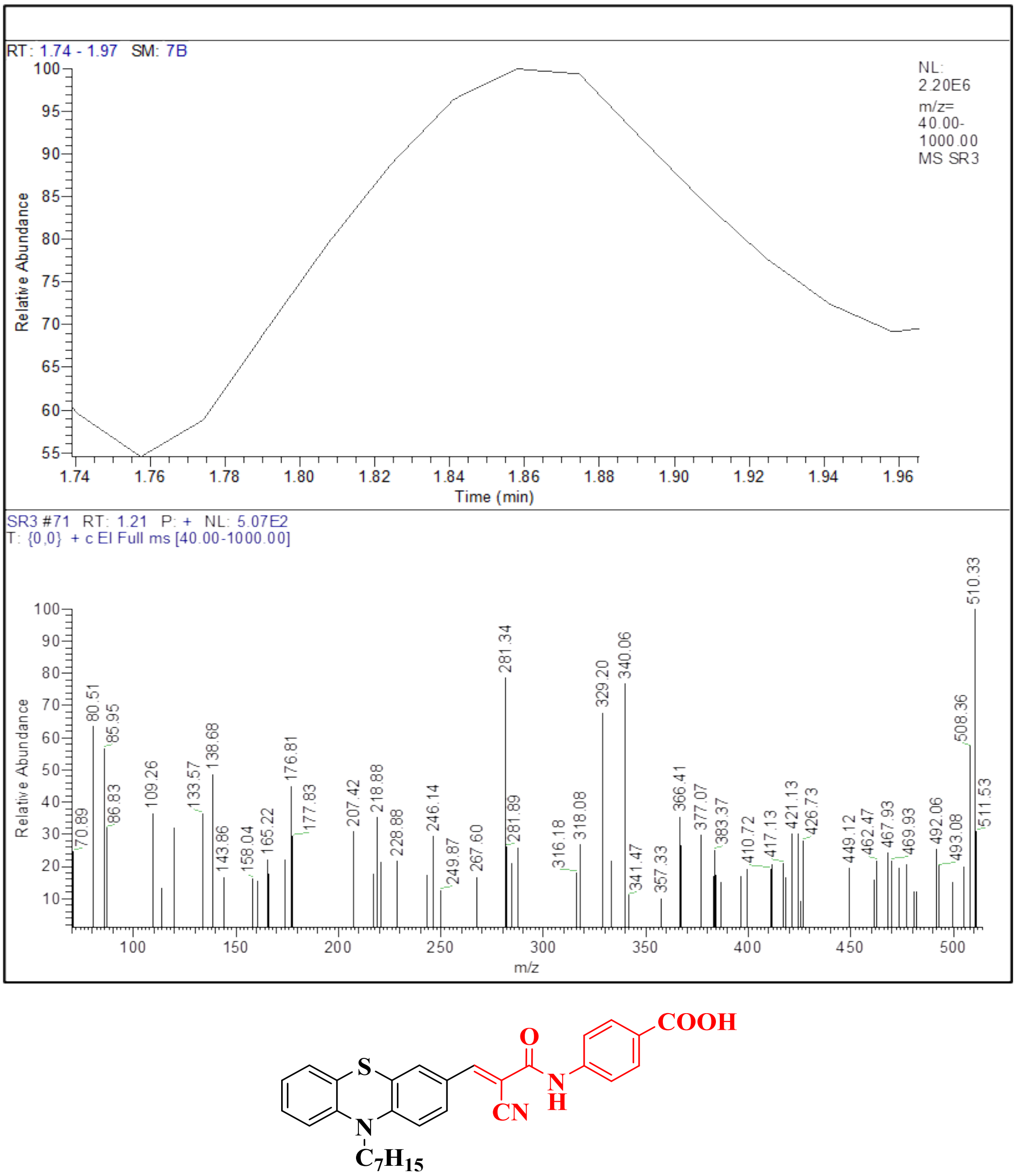

Figure (S5): Mass spectrum of sensitizer SR1.

m/z	Intensity	Relative
70.89	125.6	24.78
80.51	322.6	63.67
85.95	288.8	56.99
86.83	162.7	32.10
109.26	185.6	36.62
114.08	68.1	13.44
119.65	162.3	32.02
133.57	185.7	36.65
138.68	246.7	48.67

143.86	85.1	16.79
158.04	83.7	16.52
160.48	80.1	15.81
165.22	112.7	22.23
166.16	89.9	17.73
173.69	112.3	22.15
176.81	227.9	44.96
177.83	149.7	29.54
207.42	157.3	31.04
217.09	90.1	17.78
218.88	180.0	35.52
220.75	110.0	21.70
228.88	110.7	21.84
243.48	87.5	17.26
246.14	149.7	29.54
249.87	65.3	12.89
267.60	85.6	16.89
281.34	400.0	78.93
281.89	133.1	26.26
284.69	106.9	21.10
287.76	132.0	26.05
316.18	92.5	18.26
318.08	137.2	27.07
329.20	344.1	67.90
333.13	111.6	22.02
340.06	390.1	76.98
341.47	56.4	11.13
357.33	52.3	10.31
366.41	180.5	35.62
366.94	135.5	26.73
377.07	151.7	29.94
382.87	86.9	17.15

383.37	128.4	25.34
384.34	89.7	17.71
386.90	76.9	15.18
396.19	87.6	17.28
399.16	97.7	19.28
399.73	86.4	17.05
410.72	98.5	19.44
411.62	105.9	20.89
417.13	107.7	21.26
418.25	84.5	16.68
421.13	154.0	30.39
424.33	153.7	30.33
425.32	47.2	9.31
426.73	143.2	28.26
449.12	99.9	19.71
461.32	82.0	16.18
462.47	112.0	22.10
467.93	123.2	24.31
469.93	112.0	22.10
473.38	99.7	19.68
477.04	104.8	20.68
480.59	61.7	12.18
482.28	62.0	12.23
492.06	129.2	25.49
493.08	102.9	20.31
499.52	78.4	15.47
505.05	102.1	20.15
508.36	290.6	57.35
510.33	506.8	100.00
511.53	157.9	31.15

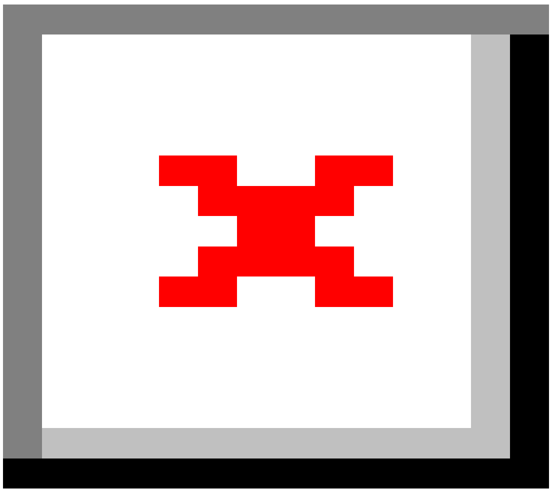

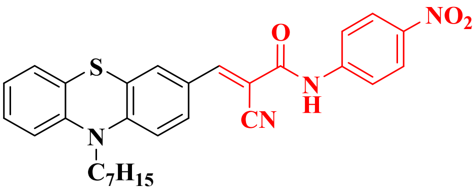

Figure (S6): IR spectrum of senstizer SR2.

https://drive.google.com/drive/folders/1f8WLWHEN9fHwhymClPLbnk6v8TinhCBx?usp=sharing

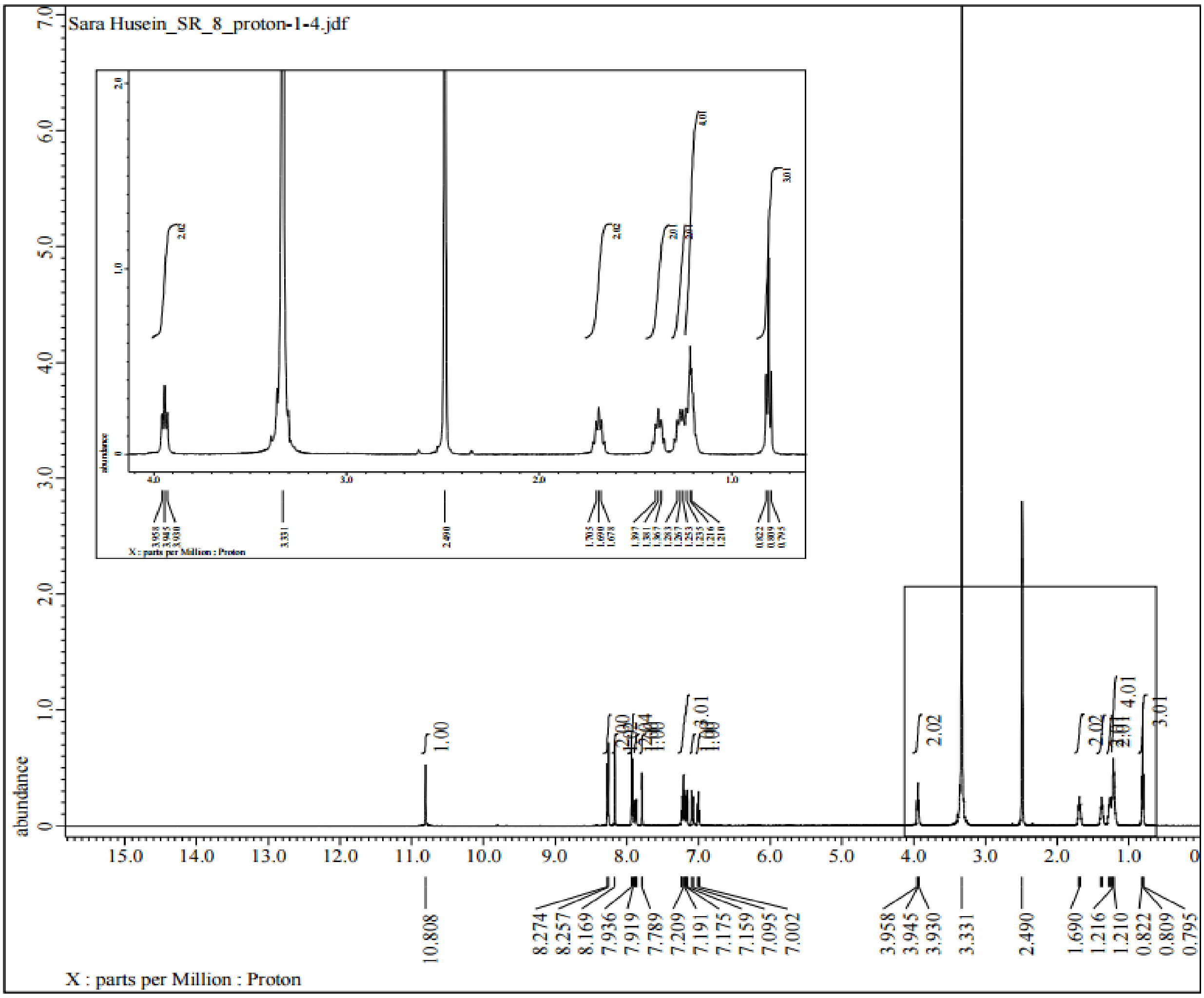

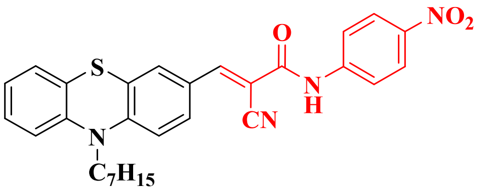

Figure (S7): ^{1}H NMR spectrum of sensitizer SR2.

https://drive.google.com/drive/folders/1jOIXmyHxDp-kELdsuwHYx-ZR-LK4bh5d?usp=sharing

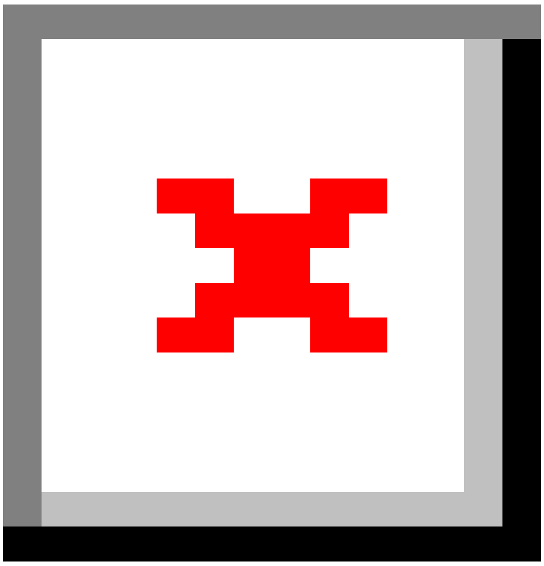

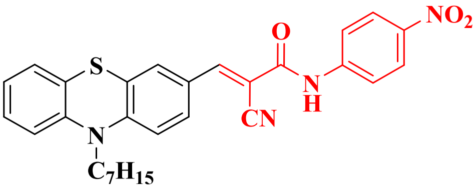

Figure (S8): ^{13}C NMR spectrum of sensitizer SR2.

https://drive.google.com/drive/folders/16PetSCwR91Hl_fwLXT1ZTxarlP5ZtvMv?usp=sharing

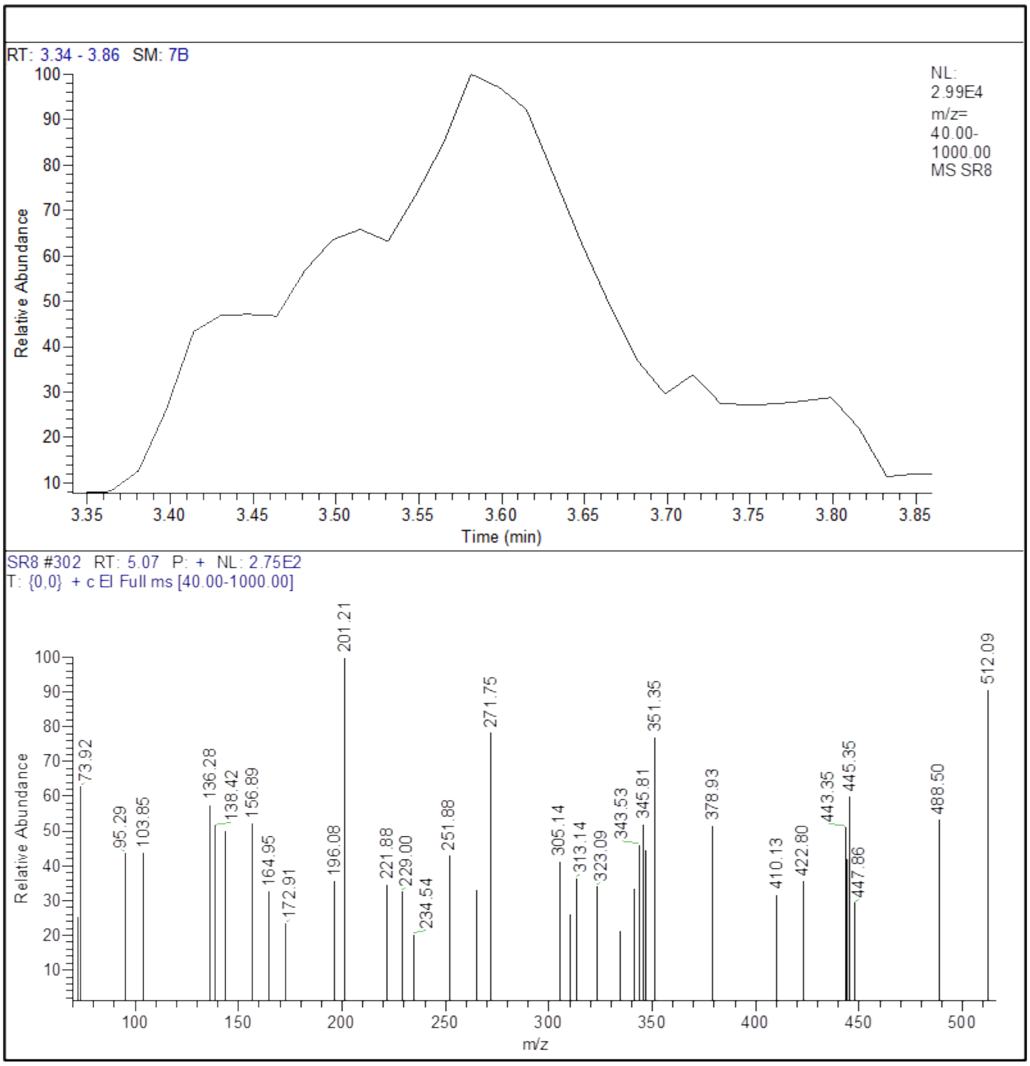

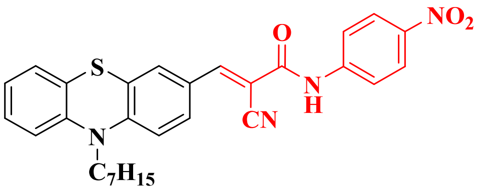

Figure (S9): Mass spectrum of sensitizer SR2.

m/z	Intensity	Relative
72.31	69.6	25.34
73.92	172.7	62.86
95.29	120.1	43.74
103.85	120.3	43.79
136.28	158.3	57.62
138.42	141.2	51.41
143.81	137.3	50.00
156.89	143.5	52.23

164.95	89.5	32.57
172.91	64.4	23.45
196.08	97.9	35.63
201.21	274.6	100.00
221.88	95.3	34.71
229.00	89.9	32.72
234.54	55.9	20.34
251.88	118.3	43.06
265.06	91.1	33.16
271.75	215.2	78.35
305.14	113.5	41.31
310.07	71.3	25.97
313.14	100.4	36.55
323.09	94.0	34.22
334.52	58.9	21.46
341.00	91.7	33.40
343.53	126.7	46.12
345.81	142.8	51.99
346.81	122.4	44.56
351.35	212.0	77.18
378.93	141.9	51.65
410.13	87.5	31.84
422.80	97.6	35.53
443.35	140.3	51.07
443.86	115.2	41.94
445.35	164.5	59.90
447.86	80.5	29.32
488.50	146.3	53.25
512.09	249.2	90.73

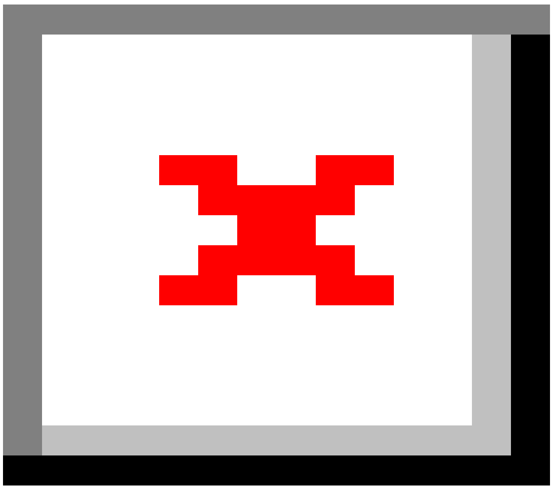

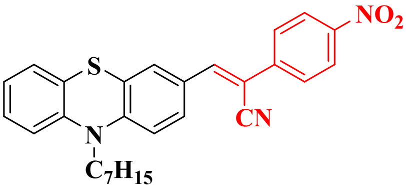

Figure (S10): IR spectrum of sensitizer SR3.

https://drive.google.com/drive/folders/1fLQotjZ4oYFCKDn07IduxbBugZG8qOJy?usp=sharing

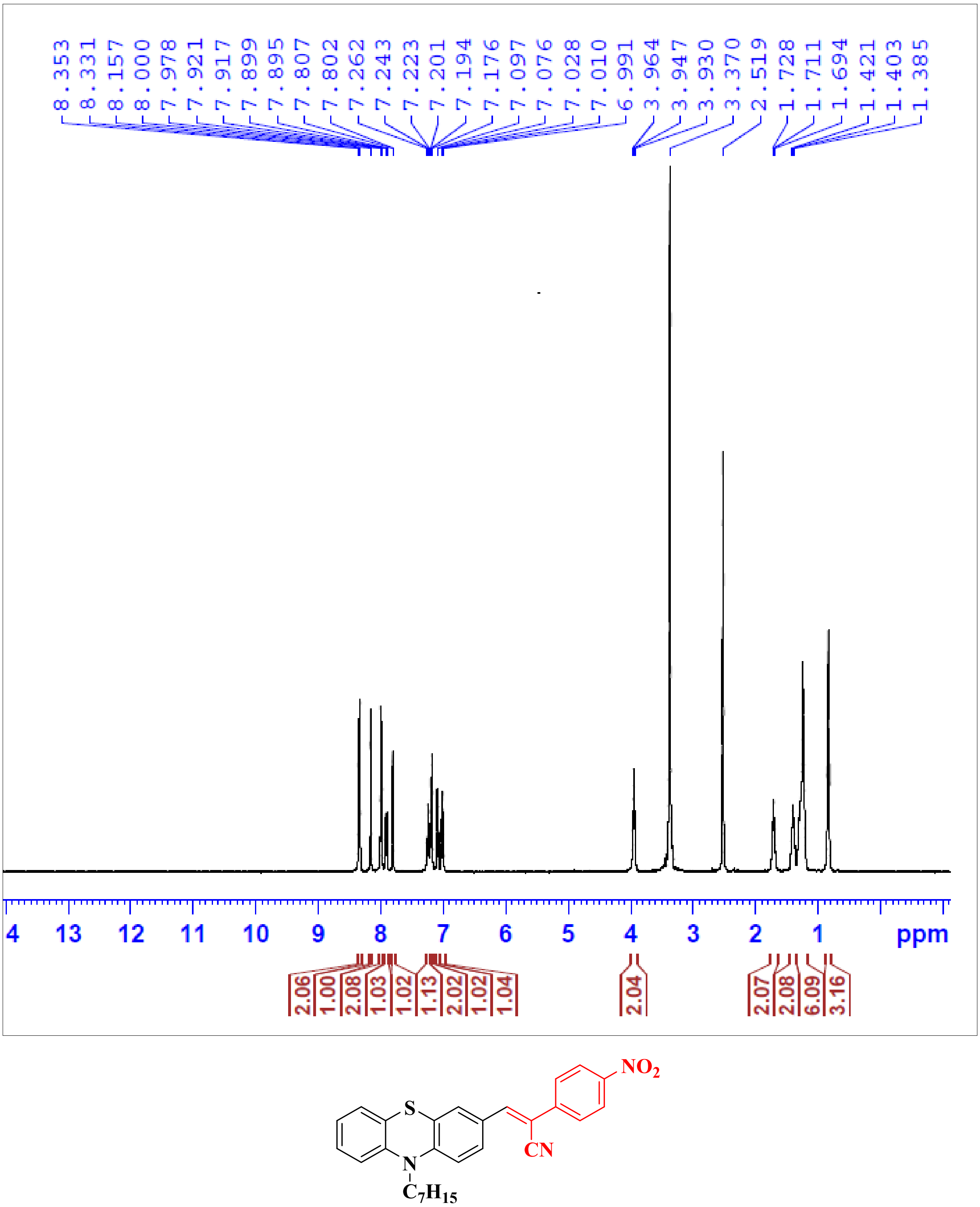

Figure (S11-A): ^{1}H NMR spectrum of sensitizer SR3.

https://drive.google.com/drive/folders/1YRaE10YPZ12dN0J6wiVAHacwnuO_rtii?usp=sharing

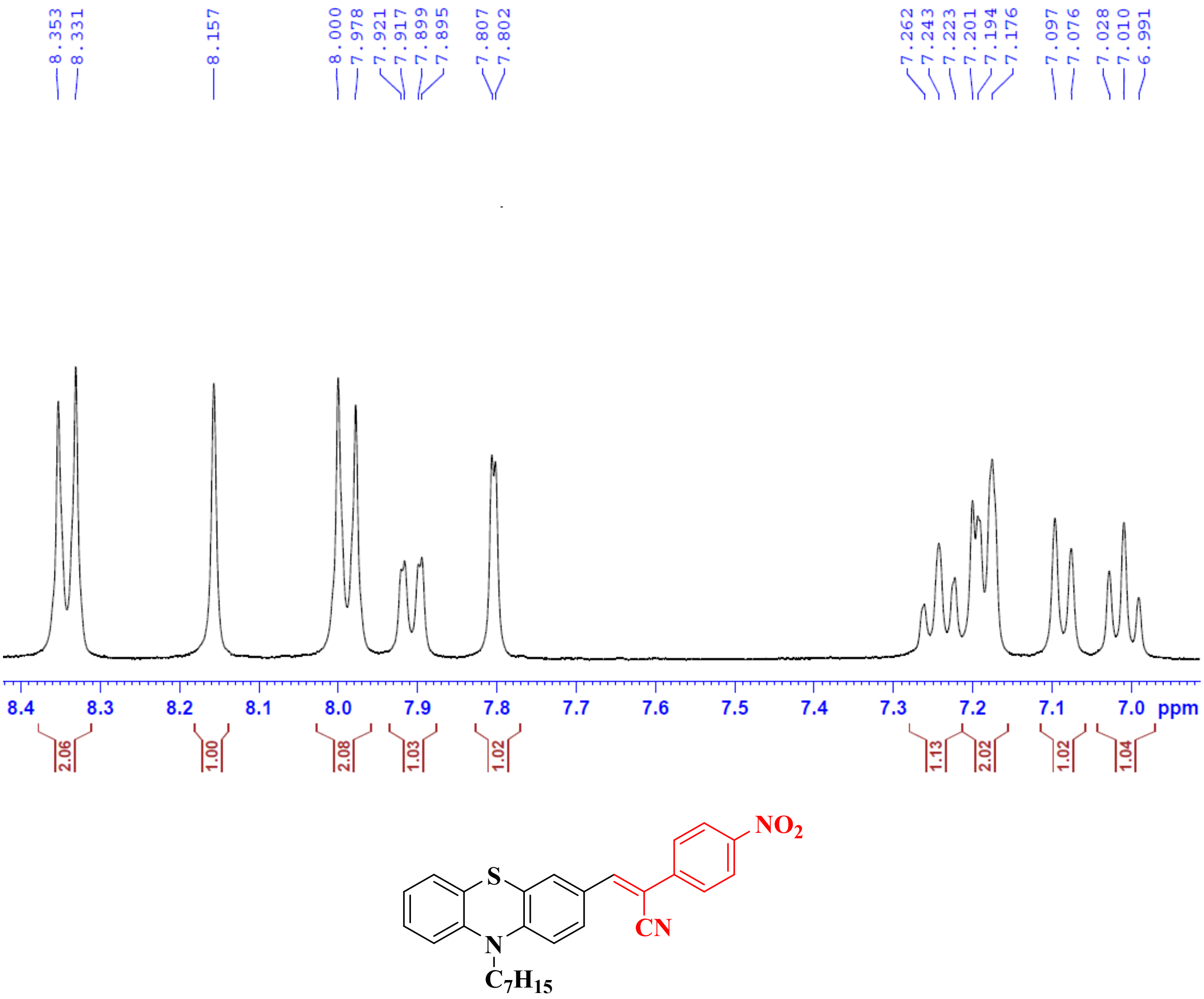

Figure (S11-B): ^{1}H NMR spectrum of sensitizer SR3.

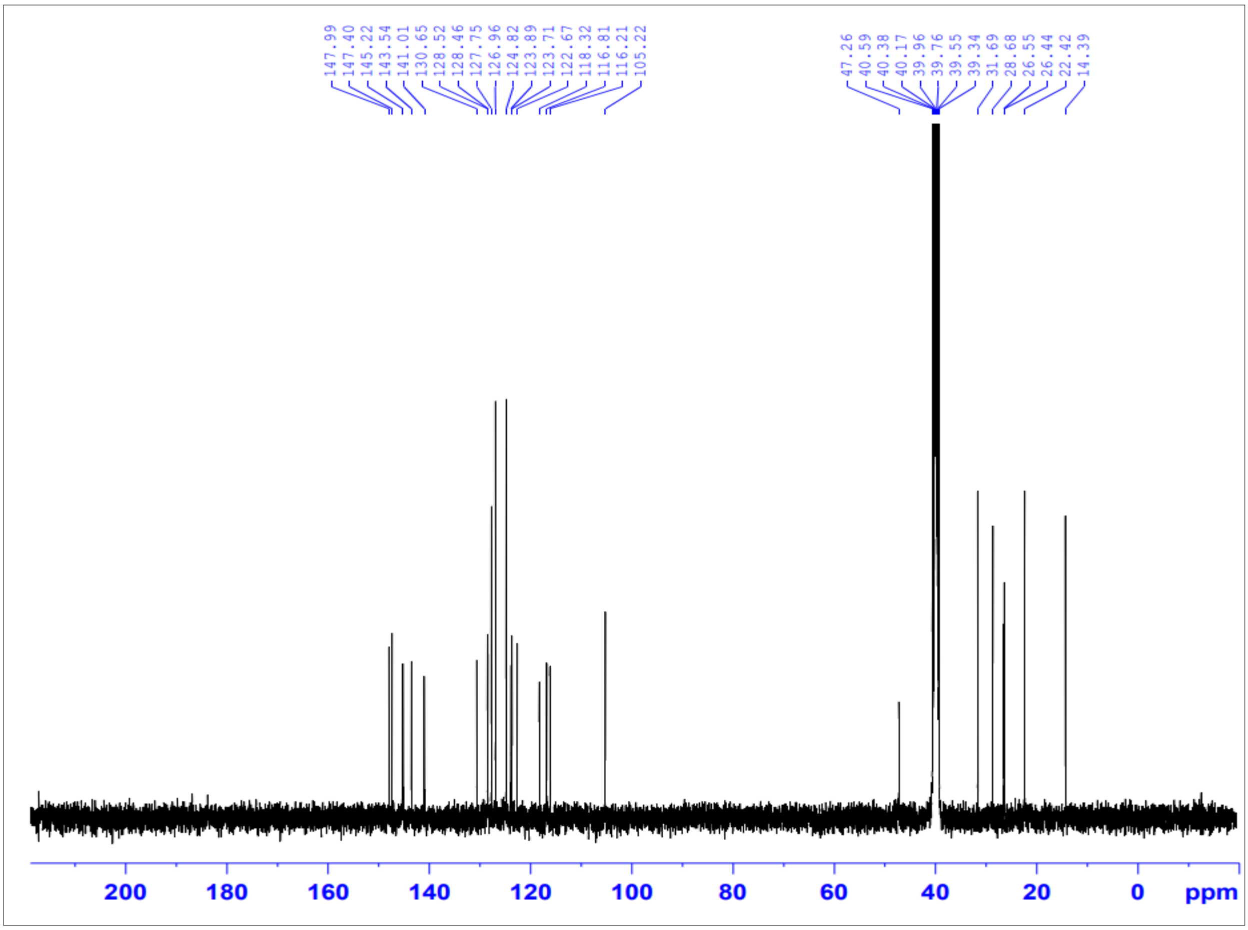

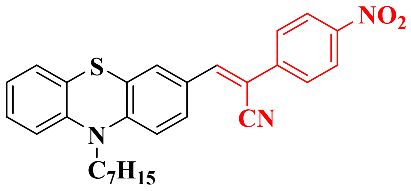

Figure (S12): ^{13}C NMR spectrum of sensitizer SR3.

https://drive.google.com/drive/folders/18NIj_ZOVYLnP1nn_4BivH3O1yBmJ1Rx4?usp=sharing

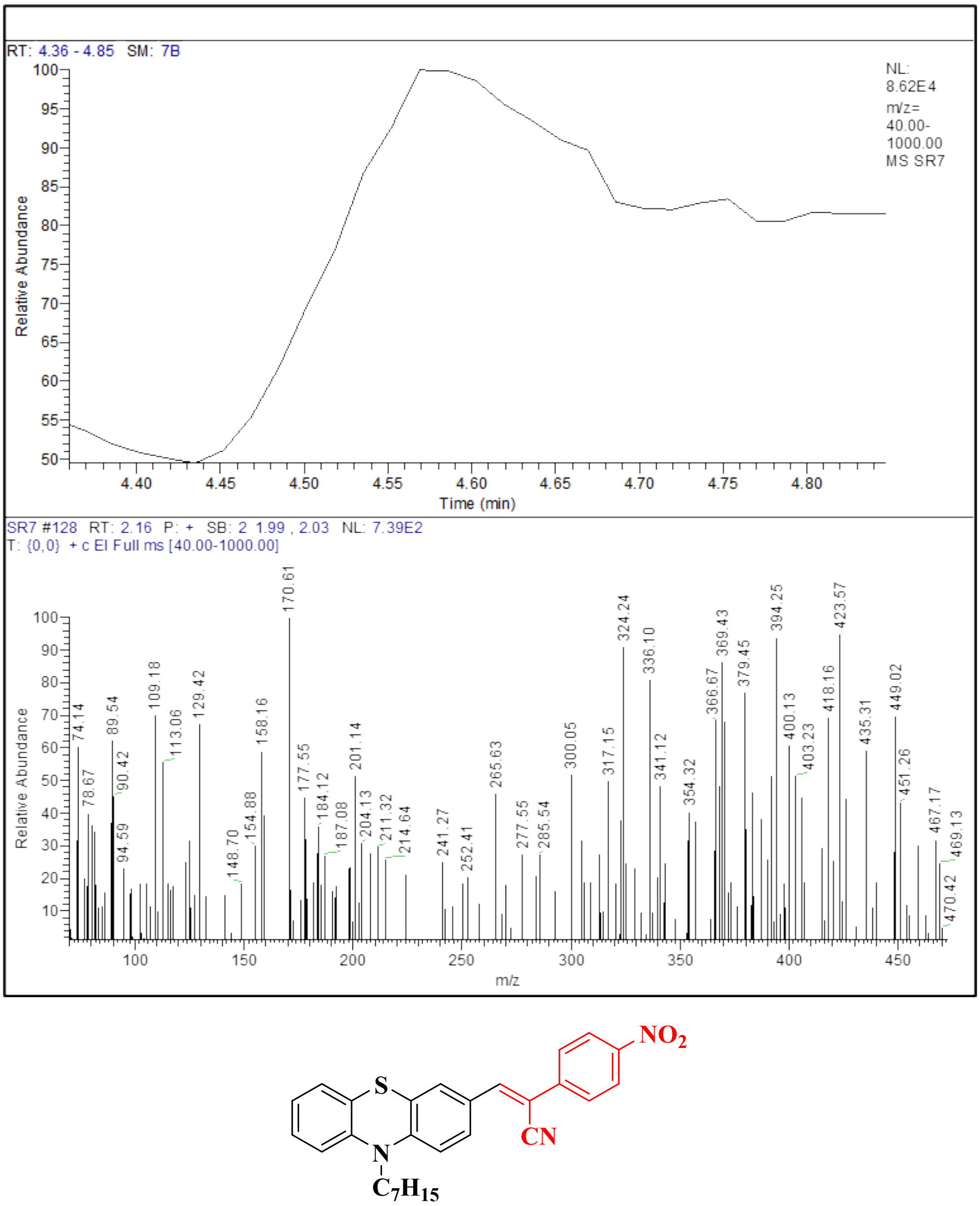

Figure (S13): Mass spectrum of sensitizer SR3.

m/z	Intensity	Relative
70.29	34.8	4.71
71.10	14.8	2.00
73.38	235.3	31.86
74.14	445.8	60.35
76.61	148.4	20.09
78.06	131.2	17.76
78.67	295.6	40.02
80.36	269.6	36.50
81.25	255.4	34.58

82.26	132.9	17.99
82.96	69.6	9.42
83.50	81.9	11.09
84.84	86.7	11.74
85.96	116.3	15.75
88.86	274.0	37.10
89.54	461.1	62.43
90.42	335.2	45.38
94.59	172.3	23.32
97.70	115.9	15.69
98.25	124.5	16.86
99.17	17.0	2.30
102.29	137.3	18.59
103.10	25.3	3.42
103.92	15.1	2.04
105.36	137.7	18.64
107.11	84.9	11.50
109.18	517.7	70.09
110.33	73.9	10.01
113.06	411.4	55.69
115.29	137.7	18.65
116.00	121.4	16.44
117.77	130.1	17.62
123.11	186.8	25.29
124.92	234.5	31.74
125.65	84.0	11.37
127.53	110.3	14.93
129.42	498.0	67.43
132.62	109.5	14.82
141.26	111.6	15.11
144.35	26.2	3.55
148.70	137.5	18.61

154.88	222.9	30.18
158.16	436.6	59.12
159.21	292.9	39.66
170.61	738.6	100.00
171.26	123.1	16.67
172.53	54.7	7.40
176.14	98.9	13.39
177.55	331.5	44.88
178.17	236.9	32.08
179.04	103.1	13.95
181.74	140.3	18.99
183.52	206.4	27.94
184.12	267.0	36.16
185.09	132.7	17.96
187.08	201.9	27.33
190.47	120.5	16.32
191.48	103.5	14.02
192.22	132.7	17.97
197.93	171.6	23.23
198.95	175.1	23.70
199.97	50.7	6.87
201.14	380.0	51.44
202.89	95.1	12.87
204.13	227.8	30.84
207.78	205.7	27.85
211.32	218.9	29.64
214.64	192.0	25.99
224.01	155.3	21.03
241.27	185.6	25.13
242.03	78.7	10.66
245.68	86.1	11.66
250.21	137.3	18.59

252.41	149.6	20.25
257.99	90.9	12.31
265.63	339.6	45.97
268.28	67.9	9.19
270.17	134.2	18.17
272.60	37.4	5.06
277.55	203.6	27.56
284.09	155.3	21.03
285.54	202.8	27.45
292.91	119.6	16.19
300.05	382.6	51.81
305.14	235.3	31.86
305.82	138.3	18.72
308.87	141.2	19.12
313.06	204.7	27.71
313.80	70.1	9.49
314.53	73.9	10.01
317.15	368.7	49.92
320.34	137.2	18.57
322.24	24.1	3.27
323.13	278.5	37.71
324.24	671.4	90.89
325.01	182.9	24.76
329.28	172.7	23.38
332.12	71.5	9.68
334.75	20.9	2.82
336.10	597.4	80.88
337.63	71.5	9.68
339.48	150.3	20.34
341.12	356.7	48.29
342.84	92.9	12.58
343.48	183.0	24.77

346.17	3.1	0.42
347.83	58.2	7.88
353.10	26.7	3.62
353.70	233.5	31.61
354.32	298.5	40.42
357.20	278.2	37.67
363.84	58.1	7.86
366.02	209.8	28.40
366.67	505.0	68.38
368.35	357.2	48.37
369.43	636.6	86.19
370.24	503.4	68.15
372.01	117.1	15.85
373.13	140.1	18.96
376.04	83.8	11.34
379.45	568.8	77.00
380.23	261.2	35.37
382.39	89.1	12.06
383.34	344.0	46.58
384.07	108.9	14.75
387.33	283.0	38.32
390.20	191.9	25.97
392.07	380.5	51.52
393.21	50.5	6.83
394.25	694.2	93.99
396.24	67.4	9.12
397.52	135.5	18.35
398.12	81.7	11.06
400.13	448.8	60.76
403.23	379.6	51.40
406.02	330.1	44.69
407.12	139.8	18.93

415.08	217.3	29.41
416.12	52.5	7.11
418.16	511.0	69.19
420.33	189.7	25.68
423.57	700.8	94.87
424.30	96.9	13.12
425.90	327.8	44.39
431.02	40.3	5.46
435.31	436.4	59.09
438.44	83.7	11.33
440.27	140.3	18.99
447.98	209.5	28.36
449.02	513.7	69.55
451.26	321.1	43.47
453.78	87.8	11.89
455.36	64.3	8.71
459.49	222.0	30.05
462.78	66.8	9.04
464.13	23.7	3.21
467.17	235.7	31.90
469.13	182.5	24.71
470.42	36.7	4.96

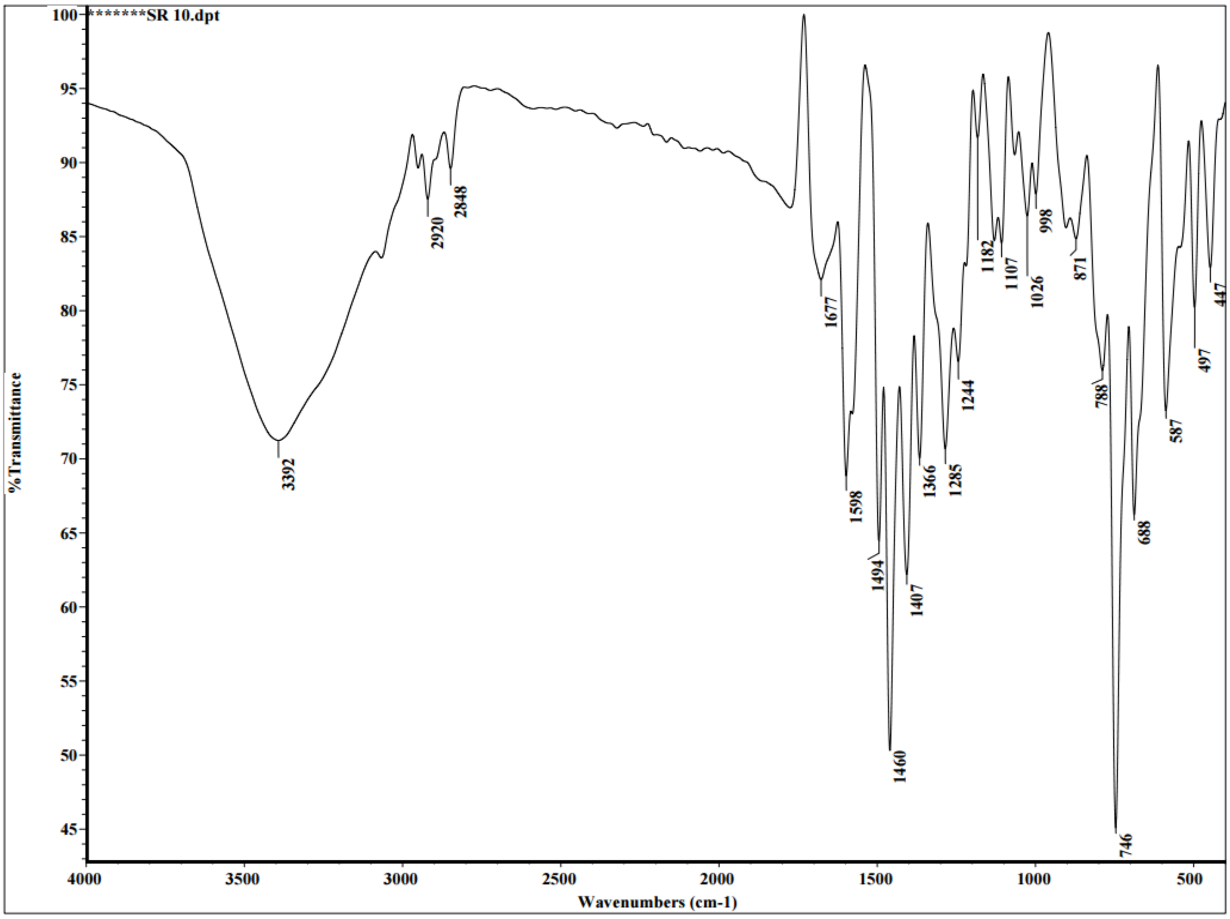

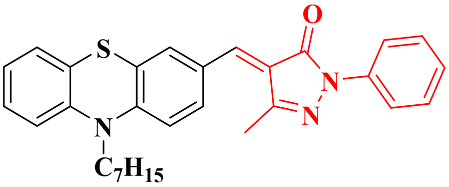

Figure (S14): IR spectrum of sensitizer SR4.

https://drive.google.com/drive/folders/1RpP5uQce9LnXenAiW71cPBBnumD4jk3m?usp=sharing

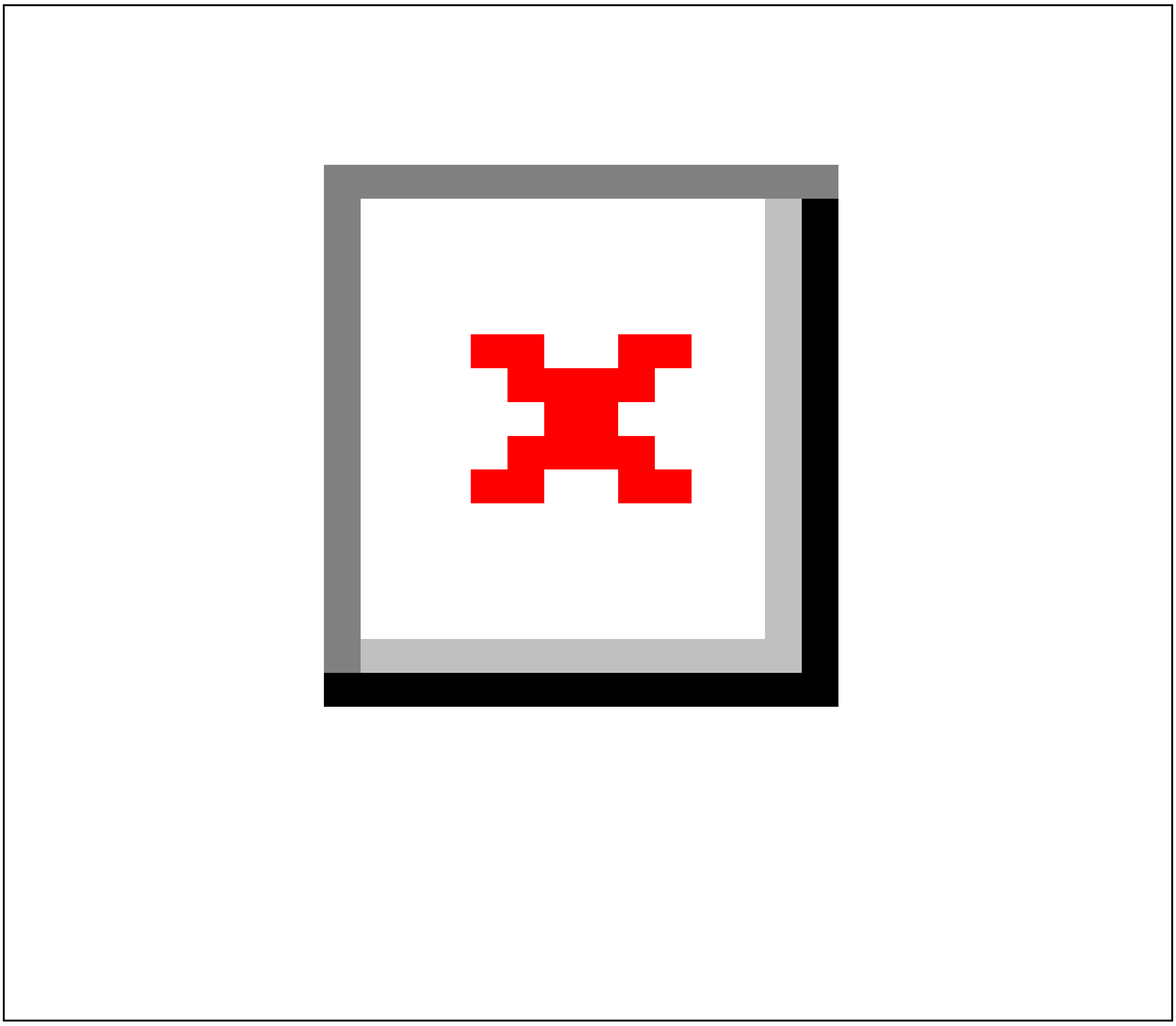

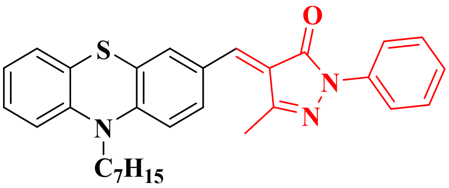

Figure (S15-A): ¹H NMR spectrum of sensitizer SR4.

https://drive.google.com/drive/folders/17Pv-YBly2hadEnzZTd0rA-y5fE5N7Nk7?usp=sharing

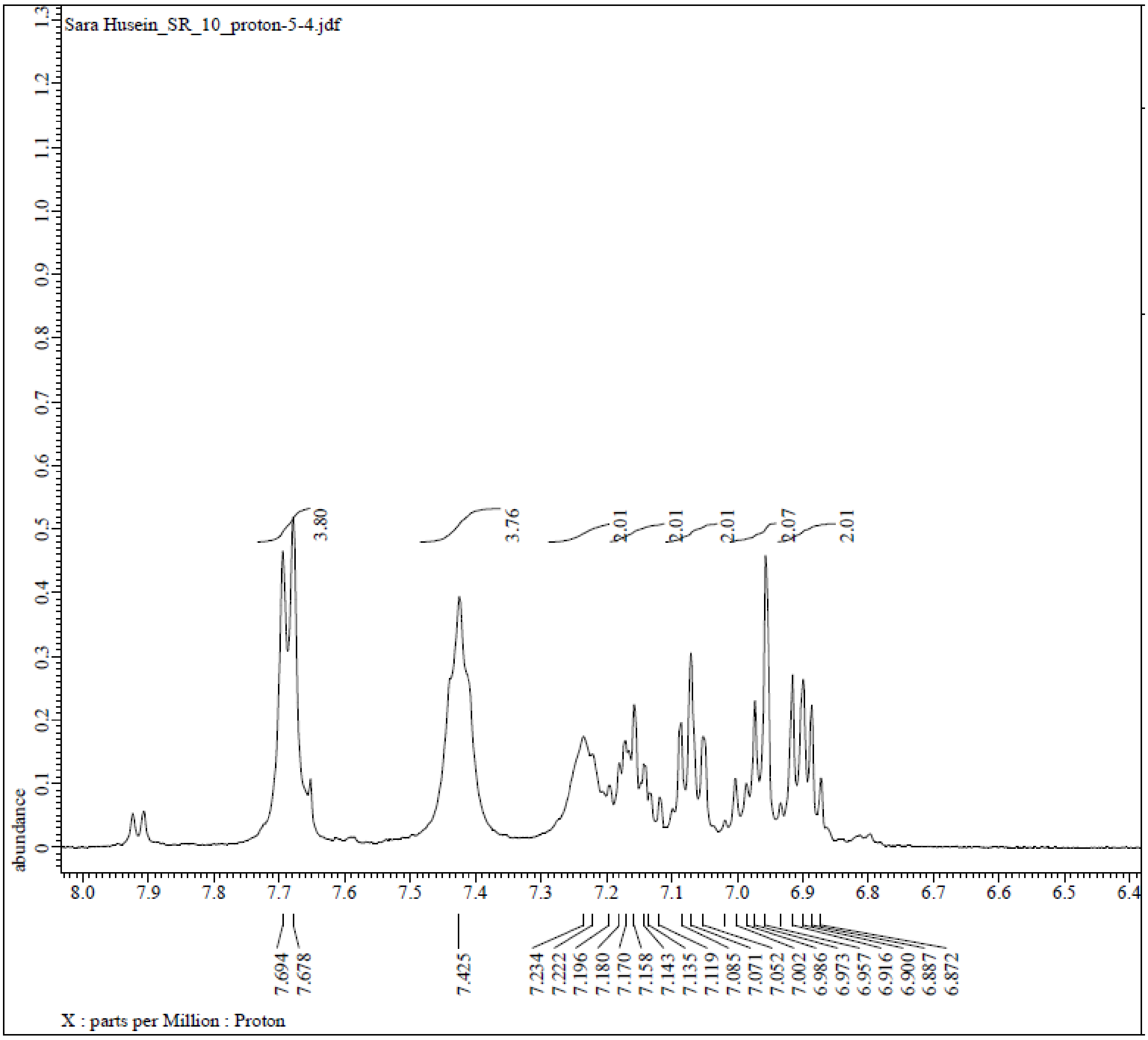

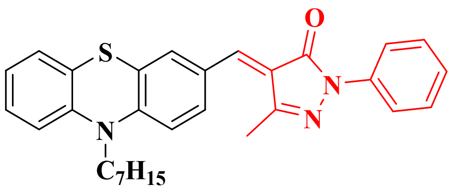

Figure (S15-B): ^{1}H NMR spectrum of sensitizer SR4.

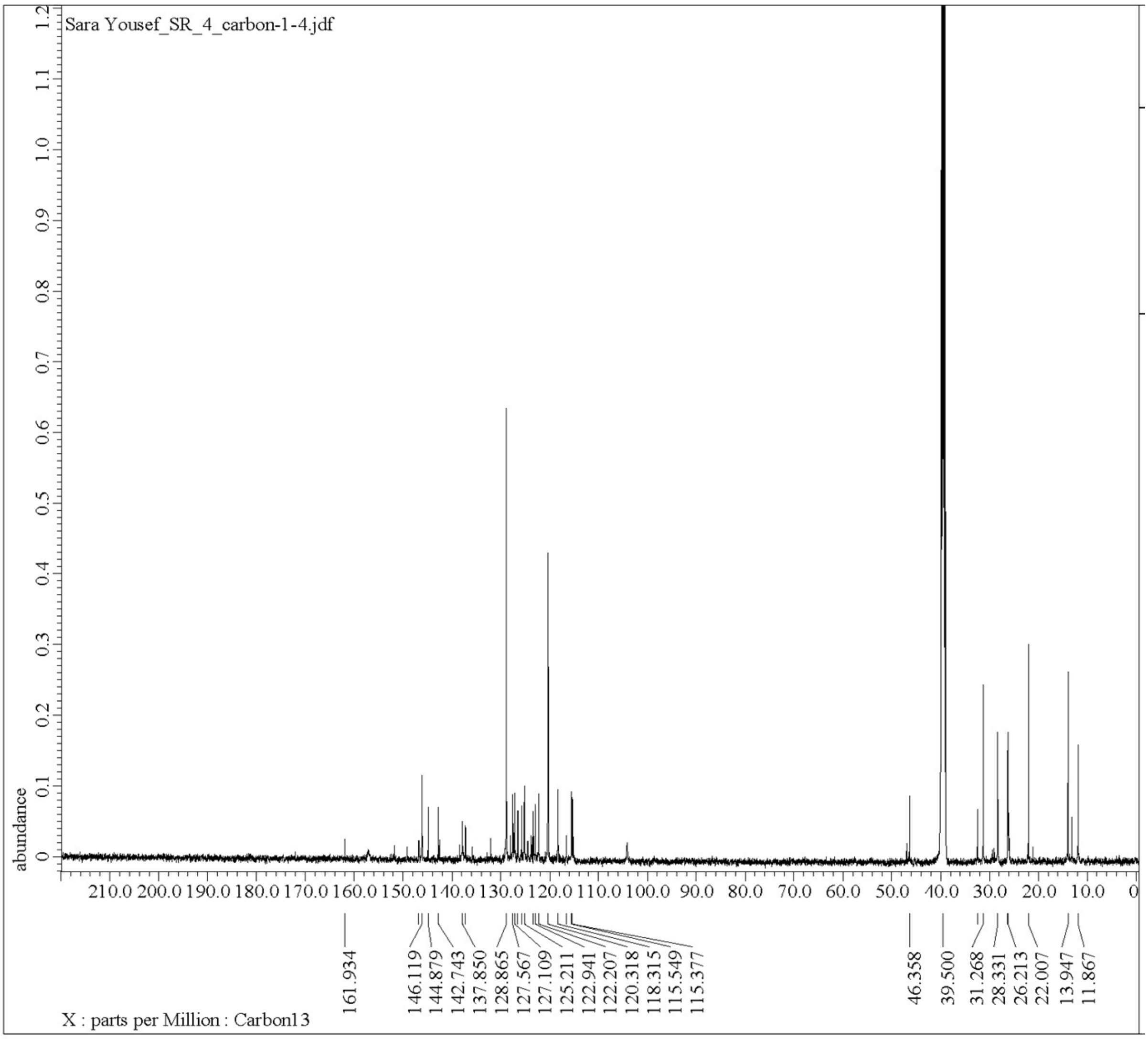

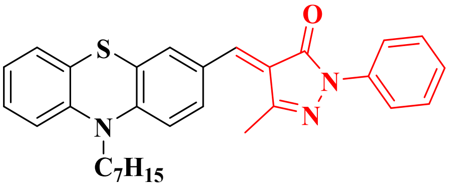

Figure (S16): ^{13}C NMR spectrum of sensitizer SR4.

https://drive.google.com/drive/folders/1VljzruZlfcAZ3Y2YmIysJ-UlPwPJjyZ5?usp=sharing

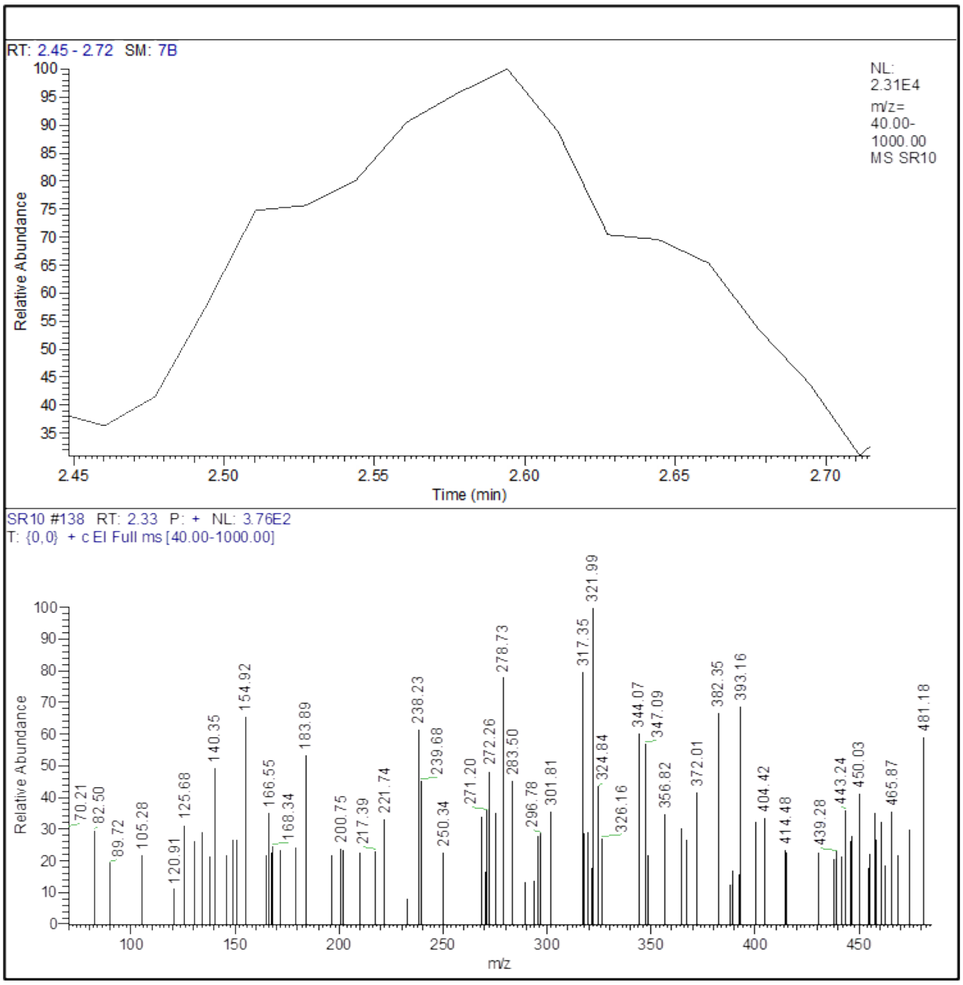

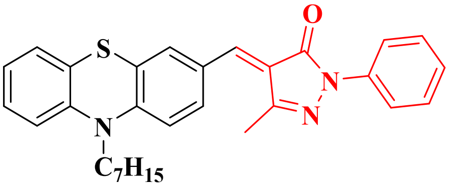

Figure (S17): Mass spectrum of sensitizer SR4.

m/z	Intensity	Relative
70.21	115.3	30.66
82.50	112.3	29.85
89.72	72.7	19.32
105.28	82.7	21.98
120.91	41.7	11.10
125.68	118.1	31.41
130.75	98.7	26.23
134.26	109.3	29.07
137.88	81.3	21.62

140.35	185.7	49.38
145.94	82.5	21.94
149.04	101.5	26.98
150.81	100.3	26.66
154.92	247.1	65.69
164.77	82.1	21.84
166.55	132.8	35.31
167.46	85.5	22.72
168.34	93.5	24.85
171.81	88.5	23.54
179.41	91.2	24.25
183.89	200.9	53.42
196.15	82.7	21.98
200.75	90.0	23.93
202.08	89.3	23.75
210.21	84.4	22.44
217.39	87.9	23.36
221.74	124.5	33.11
232.47	30.7	8.15
238.23	231.5	61.54
239.68	170.8	45.41
250.34	85.2	22.65
268.48	128.1	34.07
270.43	62.9	16.73
271.20	134.8	35.84
272.26	182.1	48.42
275.48	132.1	35.13
278.73	292.6	77.81
283.50	169.7	45.13
289.60	49.9	13.26
293.60	51.9	13.79
295.80	105.3	28.00

296.78	107.7	28.64
301.81	134.4	35.73
317.35	299.7	79.69
318.01	107.6	28.61
319.62	109.9	29.21
321.28	66.7	17.72
321.99	376.1	100.00
324.84	163.7	43.53
326.16	102.4	27.22
344.07	226.3	60.16
347.09	214.5	57.04
348.33	82.7	21.98
356.82	130.3	34.63
364.77	114.8	30.52
367.33	100.5	26.73
372.01	157.1	41.76
382.35	250.6	66.64
387.85	46.8	12.44
389.02	63.2	16.80
392.22	59.6	15.85
393.16	258.5	68.73
400.04	121.2	32.22
404.42	126.0	33.50
414.48	88.5	23.54
415.08	85.3	22.69
430.59	84.8	22.55
438.19	76.9	20.45
439.28	86.1	22.90
441.59	80.4	21.38
443.24	136.0	36.16
445.86	99.2	26.37
446.68	105.3	28.00

450.03	154.8	41.16
454.54	67.5	17.94
455.35	83.7	22.26
457.46	132.0	35.09
458.25	100.8	26.80
460.75	121.9	32.40
462.79	69.6	18.50
465.87	133.5	35.48
468.92	82.0	21.80
474.19	112.9	30.02
481.18	222.3	59.09

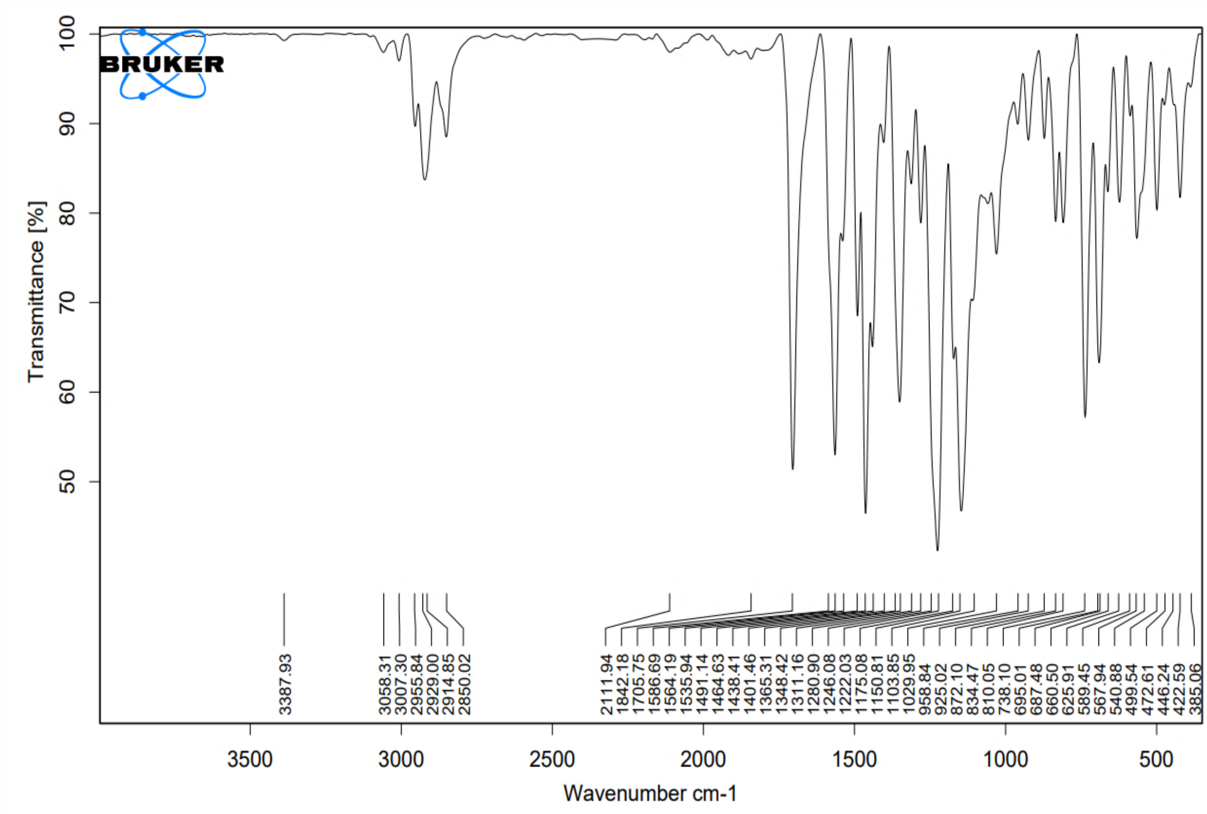

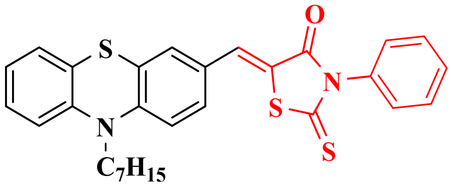

Figure (S18): IR spectrum of sensitizer SR5.

https://drive.google.com/drive/folders/1E5NLZQ2VHtgmoBcnc6Lp-8dLLl5RqA3e?usp=sharing

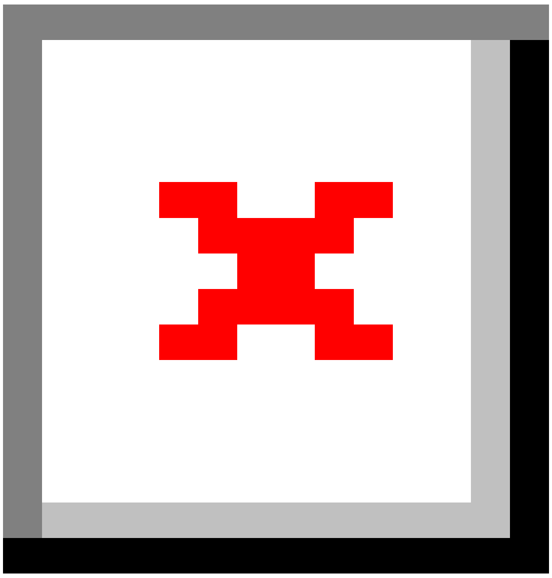

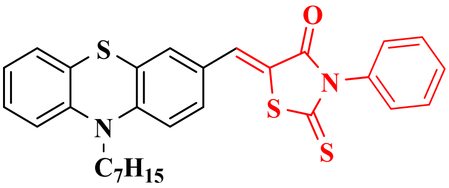

Figure (S19): ^{1}H NMR spectrum of sensitizer SR5.

https://drive.google.com/drive/folders/1jcINV8bdzIuL_yEeuLPdn2huUgIu-BQJ?usp=sharing

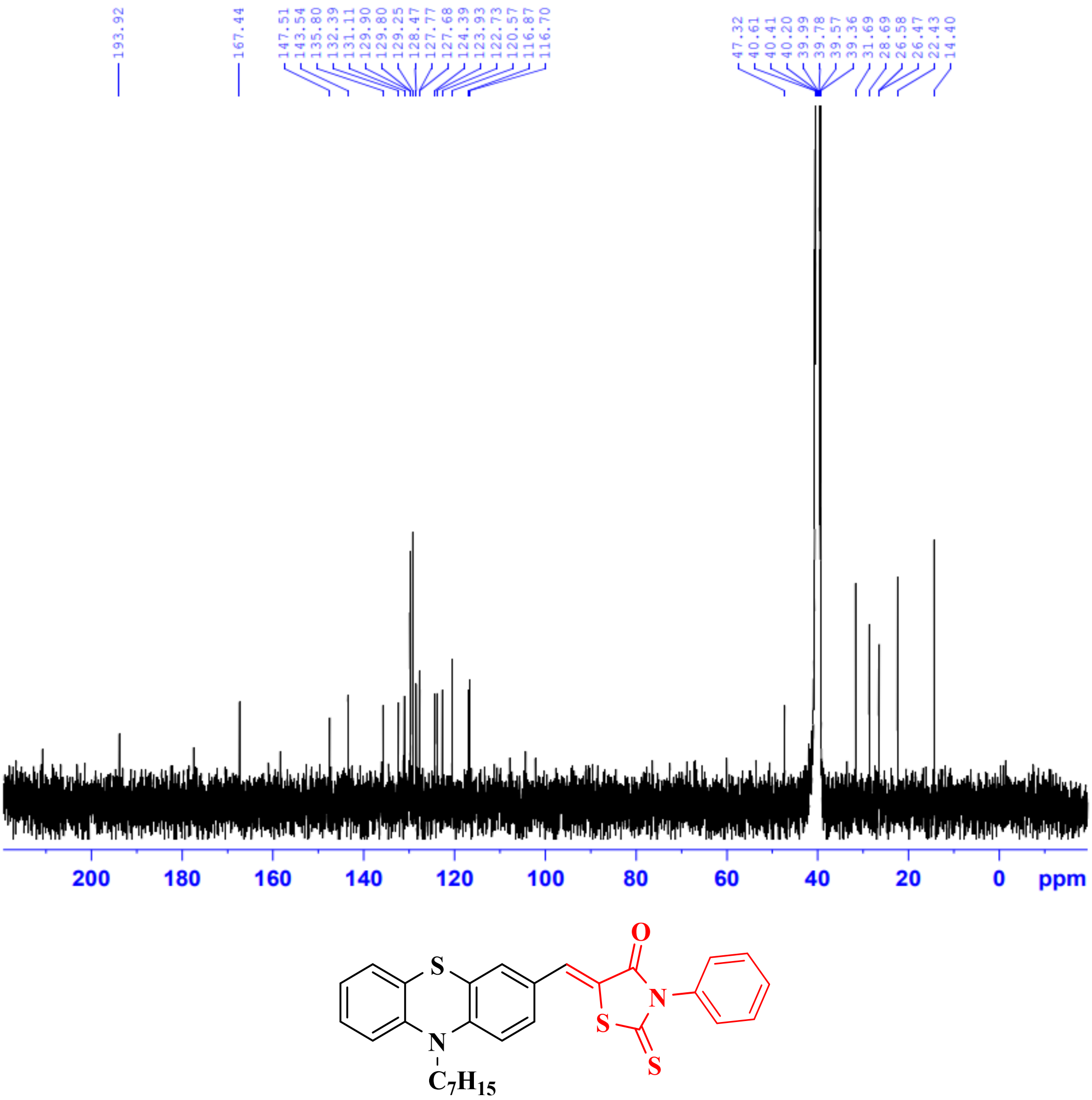

Figure (S20): ^{13}C NMR spectrum of sensitizer SR5.

https://drive.google.com/drive/folders/1ybsjREzUxP8np4LBJ-KfE_MO2F6OFcnv?usp=sharing

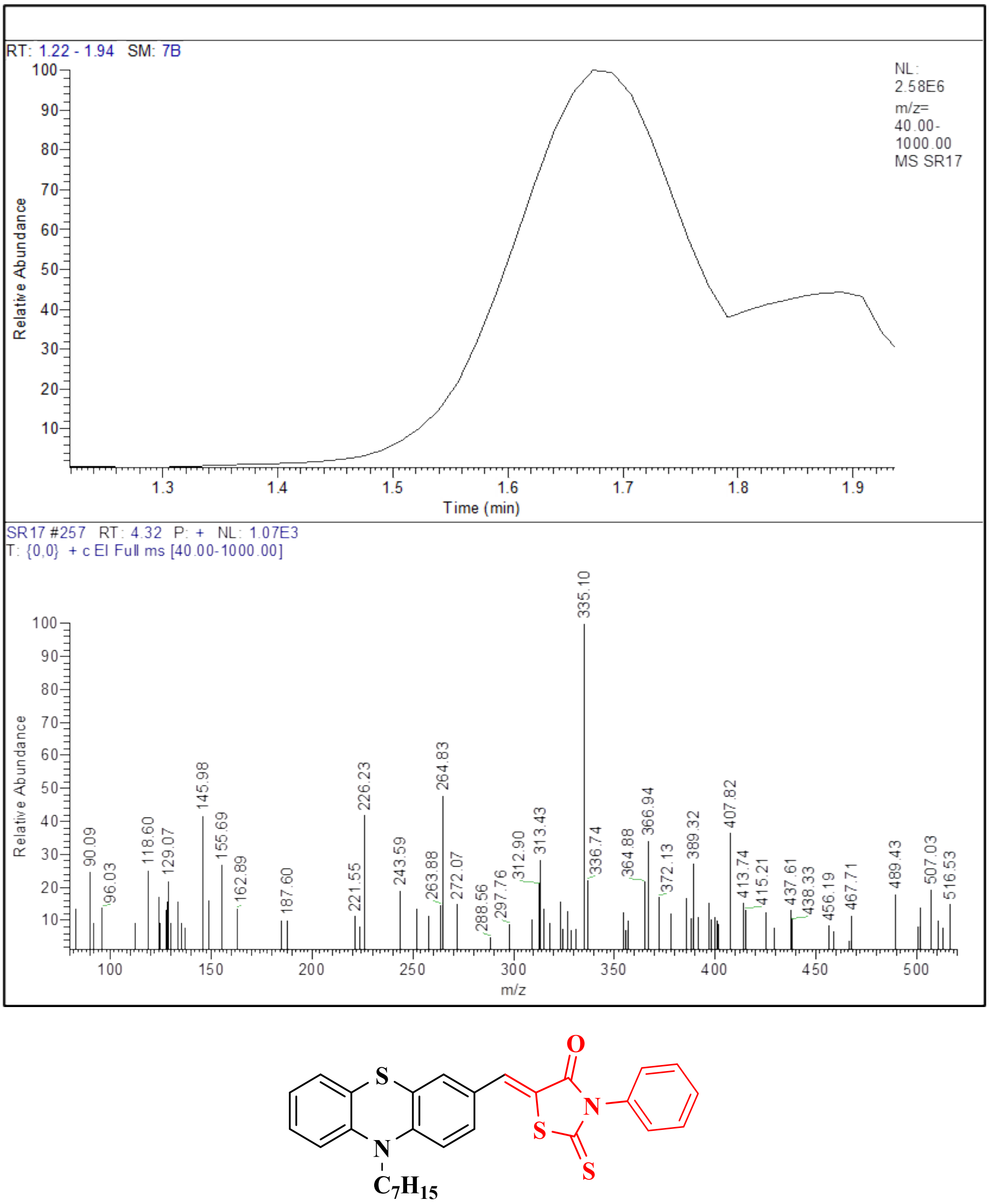

Figure (S21): Mass spectrum of sensitizer SR5.

m/z	Intensity	Relative
83.04	145.6	13.60
90.09	265.8	24.84
91.70	102.3	9.55
96.03	148.5	13.88
112.28	97.7	9.13
118.60	270.2	25.25
124.10	183.6	17.15

124.81	101.7	9.50
127.48	143.5	13.40
128.36	169.9	15.87
129.07	235.3	21.99
129.74	94.4	8.82
130.26	99.6	9.30
133.82	170.0	15.88
135.62	100.7	9.40
136.99	82.9	7.75
145.98	447.6	41.82
149.18	170.4	15.92
155.69	286.6	26.78
162.89	146.1	13.65
185.08	106.9	9.99
187.60	107.7	10.06
221.55	119.9	11.20
223.80	89.9	8.40
226.23	448.1	41.87
243.59	205.1	19.16
252.22	146.3	13.66
257.75	121.3	11.34
263.88	158.9	14.85
264.83	512.9	47.92
272.07	160.3	14.97
288.56	54.1	5.06
297.76	96.9	9.06
308.93	112.5	10.51
312.90	224.3	20.95
313.43	304.0	28.40
314.87	145.5	13.59
317.94	100.4	9.38
323.57	168.8	15.77

324.68	78.1	7.30
326.67	137.3	12.83
328.42	74.7	6.98
331.13	79.2	7.40
335.10	1070.3	100.00
336.74	240.0	22.42
354.56	132.5	12.38
355.53	77.1	7.20
357.06	106.4	9.94
364.88	232.4	21.71
366.94	366.9	34.28
372.13	183.6	17.15
378.36	128.8	12.03
385.88	181.2	16.93
387.87	116.3	10.86
389.32	290.8	27.17
391.63	120.8	11.29
396.69	166.0	15.51
398.36	110.7	10.34
399.87	118.4	11.06
400.78	107.5	10.04
401.57	94.0	8.78
407.82	390.8	36.51
413.74	164.1	15.33
415.21	141.6	13.23
425.15	134.0	12.52
429.06	83.5	7.80
437.61	140.8	13.15
438.33	108.9	10.18
456.19	89.5	8.36
458.53	71.2	6.65
466.33	43.9	4.10

467.71	123.6	11.55
489.43	194.0	18.12
500.66	87.1	8.13
501.62	149.5	13.96
507.03	208.4	19.47
510.39	106.0	9.90
512.69	81.9	7.65
516.53	160.1	14.96

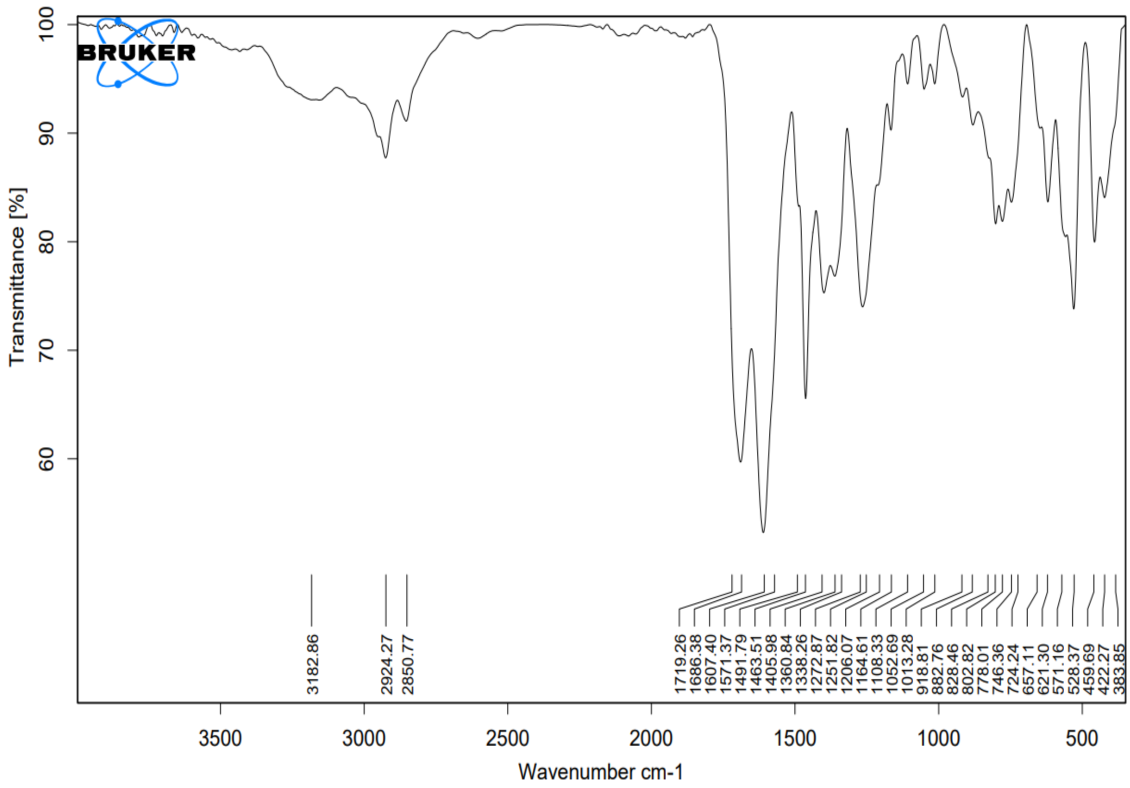

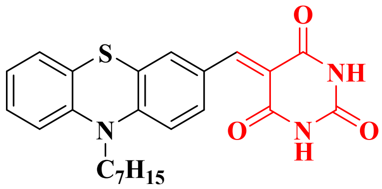

Figure (S22): IR spectrum of senstizer SR6.

https://drive.google.com/drive/folders/1_cJ8D8zbDKJa8Y1MiNv--JhE-4CgWXaI?usp=sharing

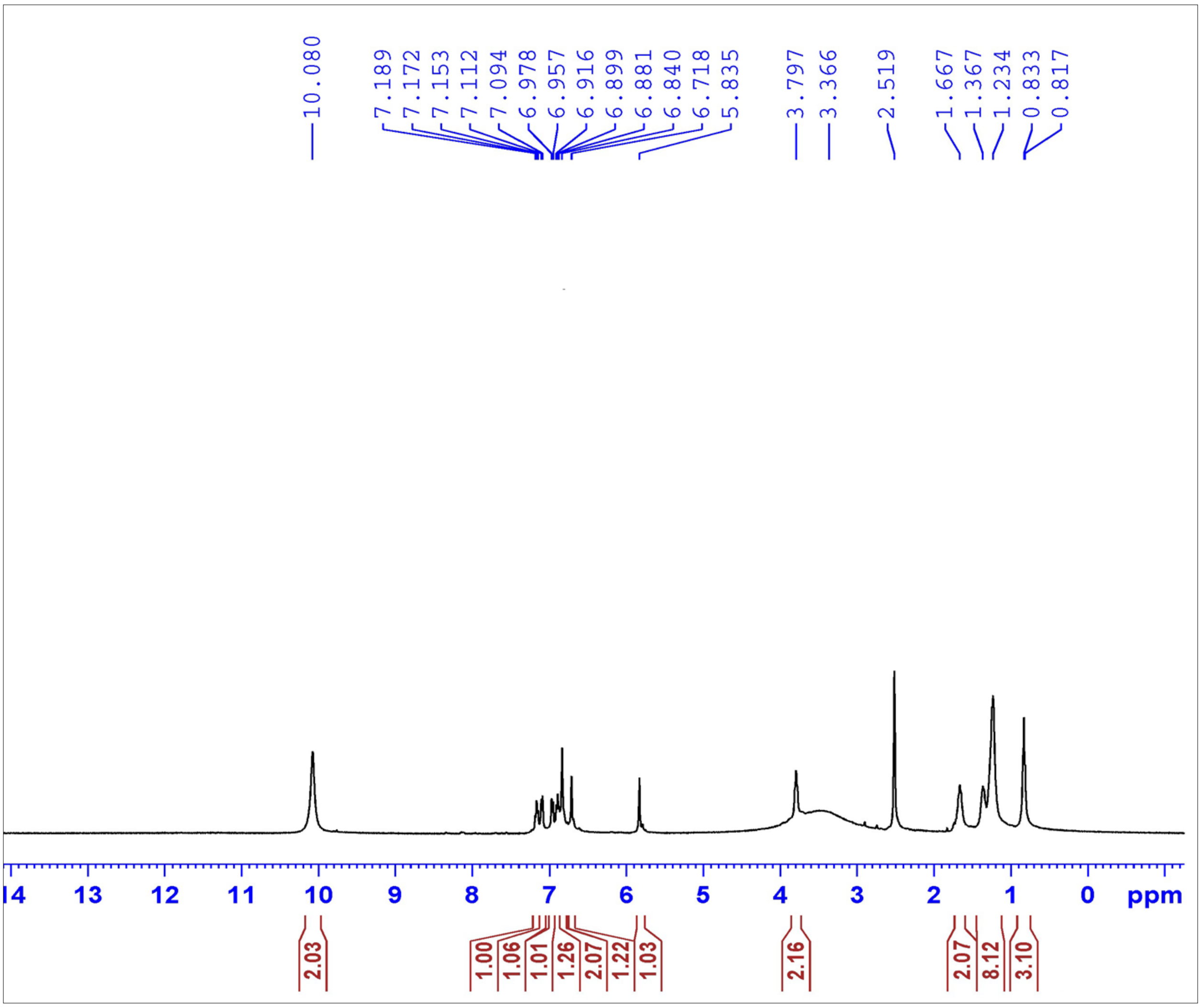

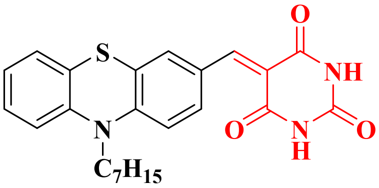

Figure (S23-A): ^{1}H NMR spectrum of sensitizer SR6.

https://drive.google.com/drive/folders/1xF8ZNMZabPWyuknb4KaRDZa9K0fPHeSL?usp=sharing

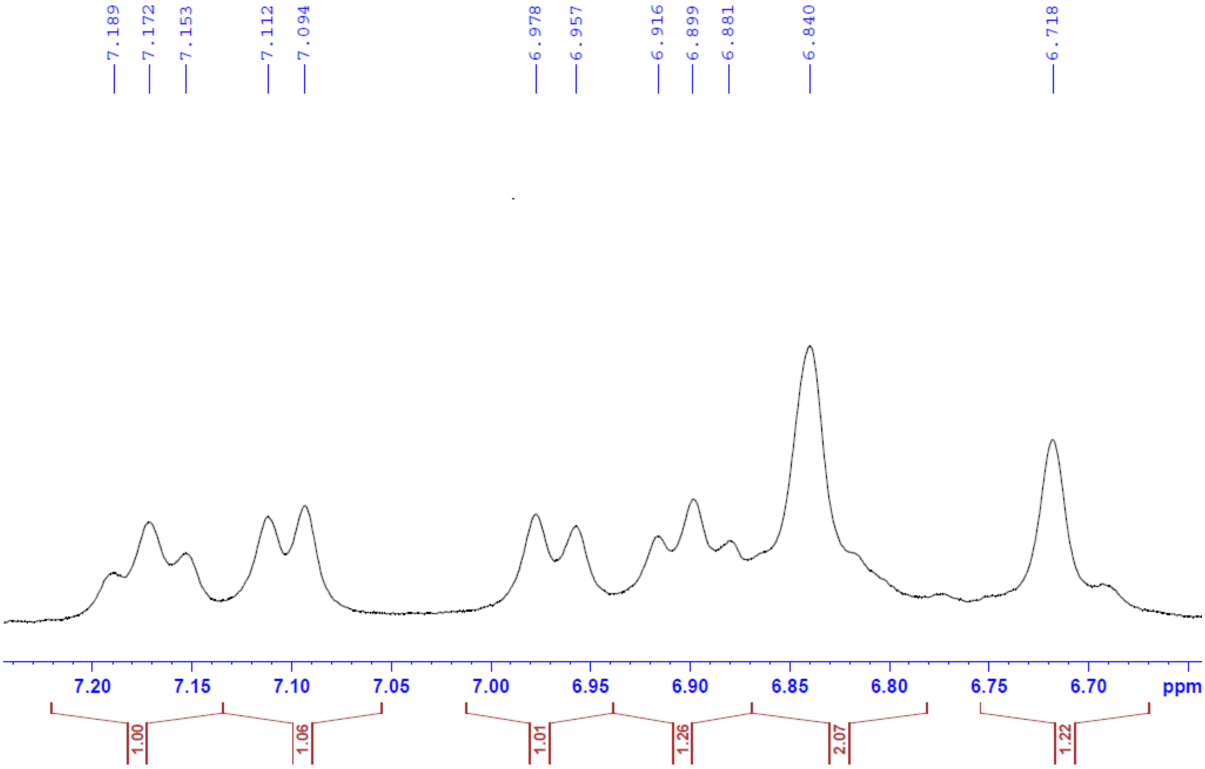

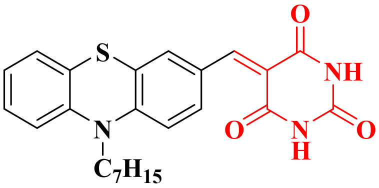

Figure (S23-B): ^{1}H NMR spectrum of sensitizer SR6.

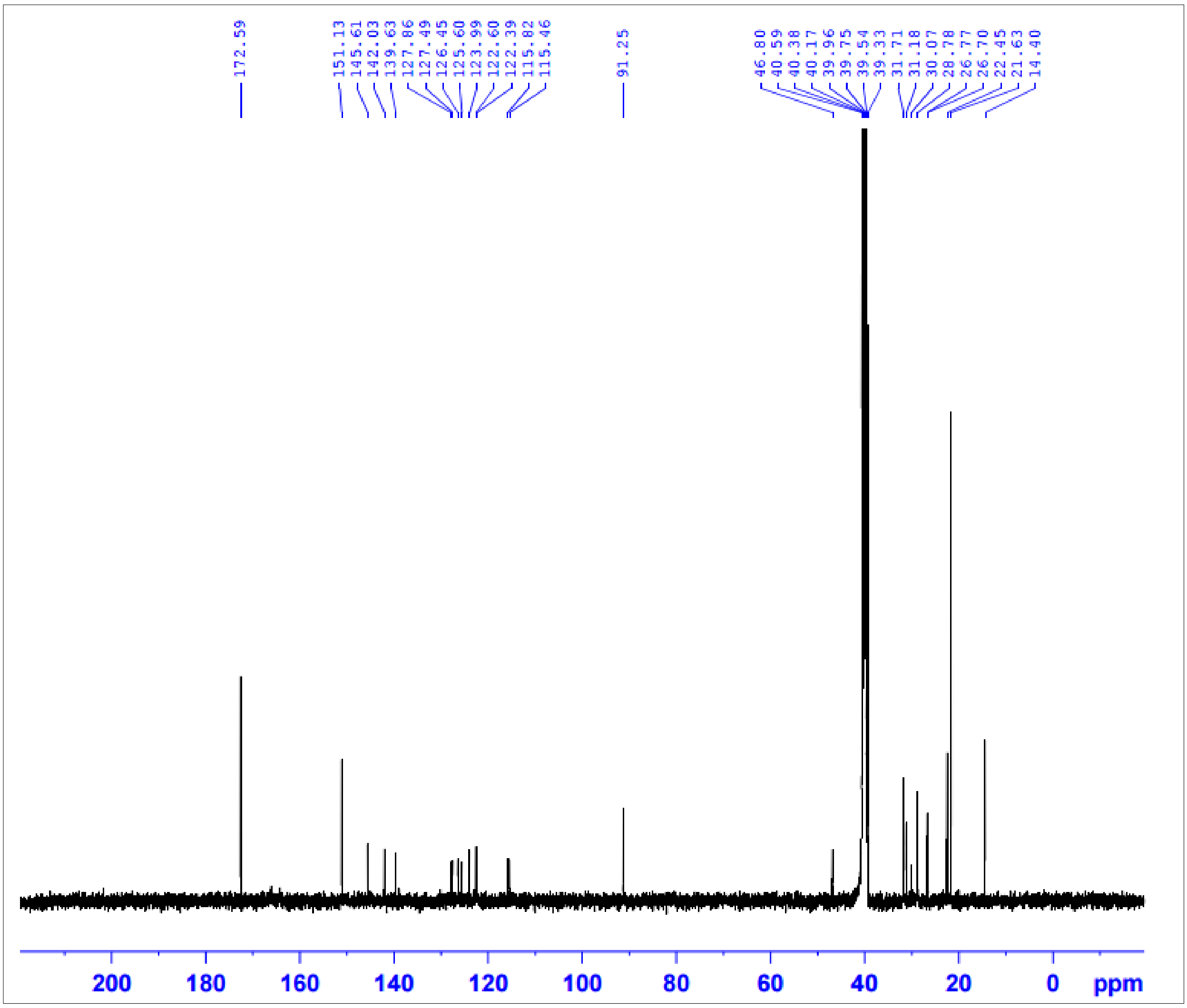

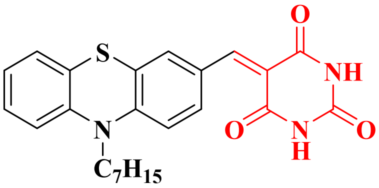

Figure (S24): ^{13}C NMR spectrum of sensitizer SR6.

https://drive.google.com/drive/folders/1Io2hXUYMqNdlAFc3vWgphbKMrp-vpush?usp=sharing

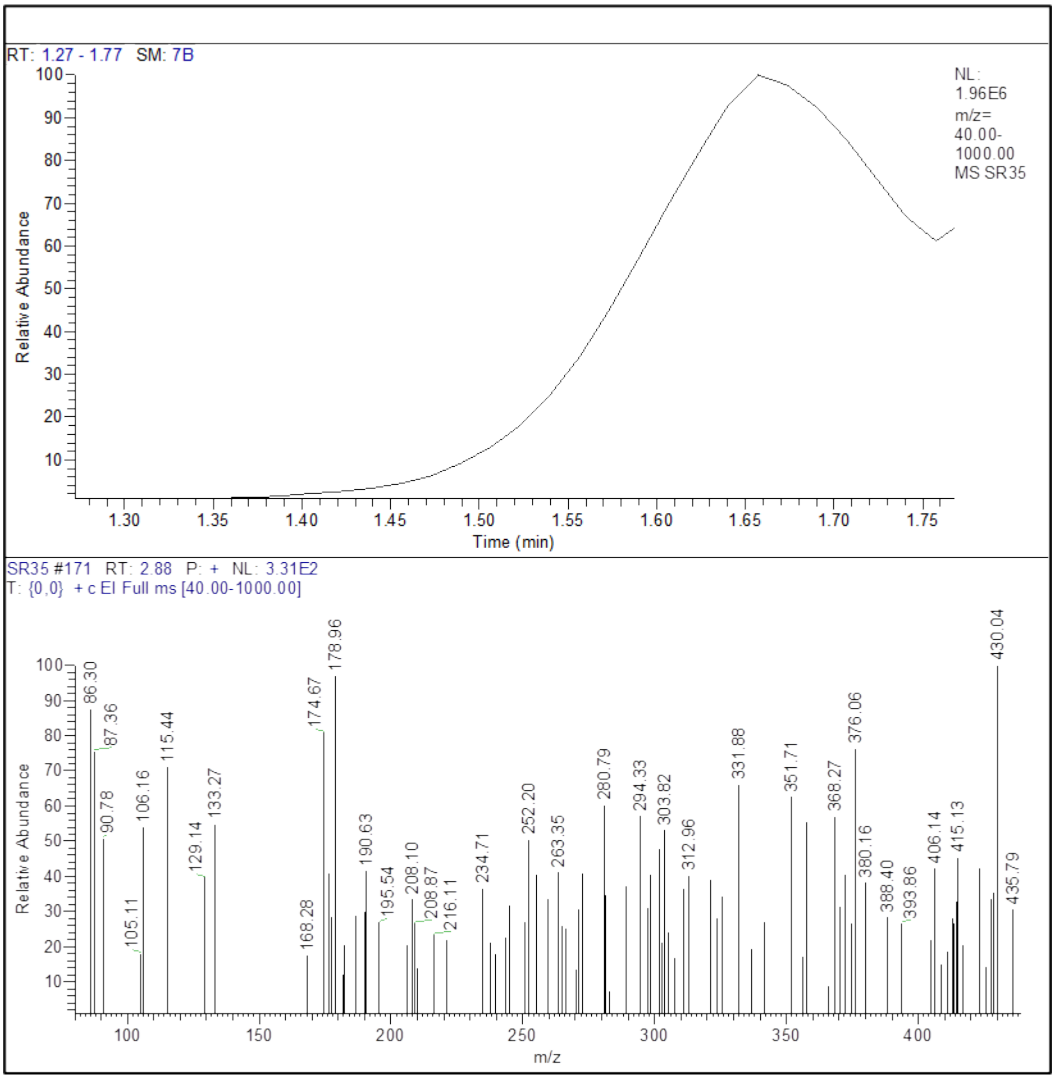

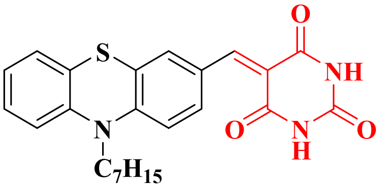

Figure (S25): Mass spectrum of sensitizer SR6.

m/z	Intensity	Relative
80.45	118.8	35.86
86.30	290.4	87.65
87.36	250.5	75.61
90.78	168.5	50.87
105.11	59.9	18.07
106.16	178.8	53.96
115.44	235.1	70.95
129.14	131.3	39.64

133.27	182.1	54.97
168.28	57.9	17.46
174.67	267.8	80.85
176.71	135.9	41.01
177.25	94.4	28.49
178.96	321.4	97.02
181.54	40.5	12.23
182.47	67.3	20.32
186.68	96.3	29.05
190.11	100.0	30.18
190.63	138.0	41.65
195.54	89.2	26.92
206.16	68.1	20.56
208.10	111.5	33.64
208.87	88.3	26.64
209.95	47.1	14.21
216.11	77.3	23.34
221.39	72.9	22.01
234.71	121.5	36.66
237.66	71.3	21.53
239.67	59.9	18.07
243.41	75.7	22.86
245.20	105.3	31.79
250.81	90.3	27.24
252.20	167.2	50.46
255.34	134.7	40.64
259.49	111.7	33.72
263.35	136.5	41.21
264.73	86.4	26.08
266.21	83.2	25.11
270.36	44.8	13.52
271.07	102.4	30.91

272.88	134.9	40.72
280.79	199.9	60.32
281.55	115.2	34.77
282.96	24.5	7.40
288.97	123.7	37.34
294.33	189.5	57.18
297.60	102.8	31.03
298.56	133.7	40.36
301.74	158.1	47.73
302.61	70.1	21.17
303.82	176.1	53.16
305.27	80.5	24.31
307.73	56.5	17.06
311.27	121.1	36.54
312.96	133.3	40.24
321.16	129.7	39.15
323.88	92.9	28.05
325.82	113.6	34.29
331.88	219.2	66.16
336.87	64.9	19.60
341.53	89.7	27.08
351.71	207.3	62.58
356.13	57.6	17.38
357.58	183.6	55.41
366.08	28.8	8.69
368.27	189.1	57.06
370.12	104.1	31.43
372.34	134.5	40.60
374.72	89.1	26.88
376.06	252.8	76.30
380.16	126.9	38.31
388.40	94.5	28.53

393.86	88.5	26.72
404.80	72.5	21.89
406.14	140.4	42.37
408.47	50.1	15.13
411.26	62.1	18.75
412.95	92.8	28.01
413.60	88.4	26.68
414.53	108.8	32.84
415.13	150.3	45.35
416.93	68.1	20.56
423.21	141.1	42.58
425.73	47.9	14.45
427.73	111.9	33.76
428.47	117.3	35.41
430.04	331.3	100.00
435.79	101.7	30.70

Photovoltaic measurements data:

1) UV data

https://docs.google.com/spreadsheets/d/1mfF2bt4Wr1WQqOZhMMiYlCBMkgkZXUAs/edit?usp=sharing&ouid=110049855877644735867&rtpof=true&sd=true

1) UV data on TiO_2

https://drive.google.com/drive/folders/1gADKCD9ABEt1Gqfx1Amq_CeGhSsP6Lsh?usp=sharing

2) I-V data for sensitizers SR-1 to SR-6

https://drive.google.com/drive/folders/1B5RXpbshlI19QUZJ5jWD-mQJ12hTBXVU?usp=sharing

3) I-V data for co-sensitizers SR1-6 + N719

https://drive.google.com/drive/folders/1i3aiqvpt0fQArIX0_nUGrvNNUht5p4a1?usp=sharing

4) IPCE data for sensitizers SR-1-6.

https://drive.google.com/drive/folders/1EAZ25SRO2LATx-Pxb_xO1zGC4XbF5mac?usp=sharing

5) IPCE data for co-sensitizers SR-1-6.

https://drive.google.com/drive/folders/15OsLLxEGf1oWt-JlO1f8Ct6yiJaCq2tm?usp=sharing

6) Impedance data for co-sensitizers SR-1-6

https://drive.google.com/drive/folders/1WCRDbj3ecMJ4-Ed-4f-UevWNyssgpauk?usp=sharing

7) Cyclic voltammetry data

https://drive.google.com/drive/folders/10WSDuNjo4pHo0uXdQ7fkS-AuPzT-J_oj?usp=sharing

Table (S1) Optical property values for **SR1-6**.

Compounds	Experimental (eV)			Theoretical (eV)		
	E_{0-0}	GSOP	ESOP	E_{0-0}	GSOP	ESOP
**SR1**	2.27	-5.81	-3.54	2.49	-5.91	-3.42
**SR2**	2.20	-5.89	-3.69	2.31	-5.85	-3.54
**SR3**	2.25	-6.02	-3.77	2.42	-6.04	-3.62
**SR4**	2.00	-5.75	-3.75	1.98	-5.63	-3.65
**SR5**	2.19	-5.90	-3.71	2.15	-6.02	-3.87
**SR6**	2.03	-5.63	-3.60	2.11	-5.73	-3.62

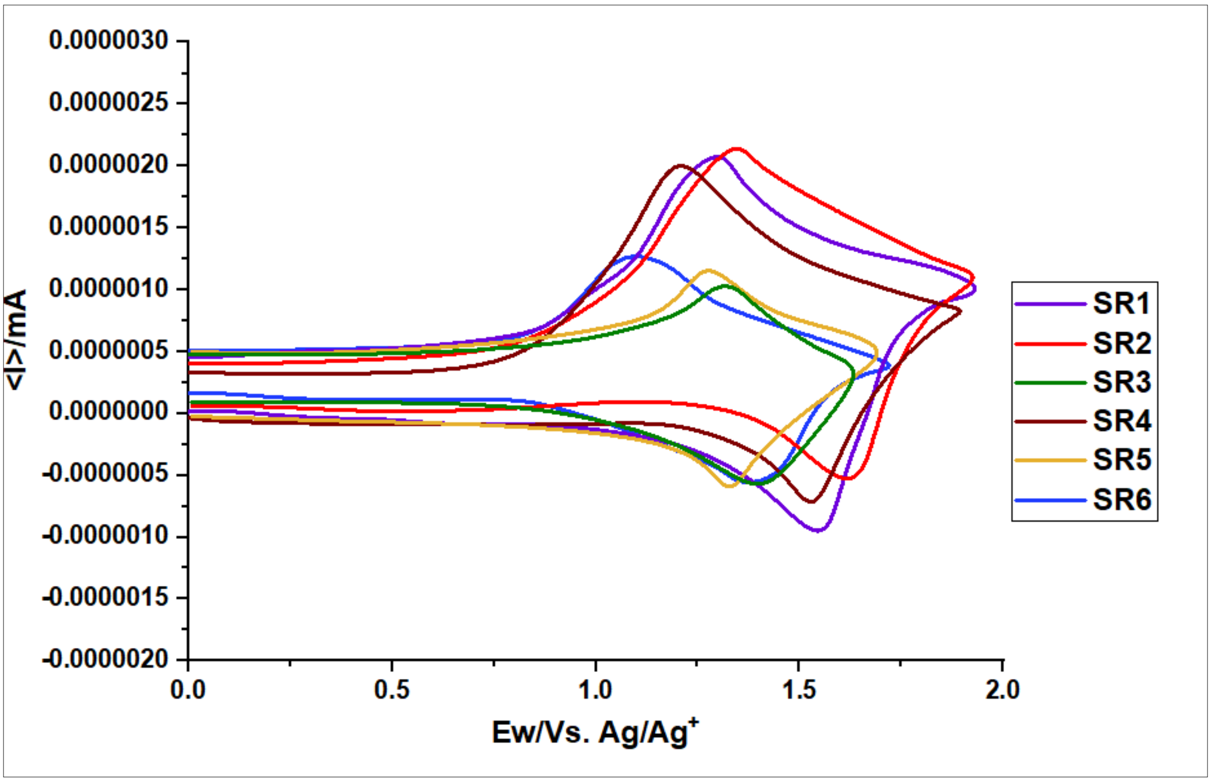

Figure (S26): Cyclic voltammograms of **SR1-6**.

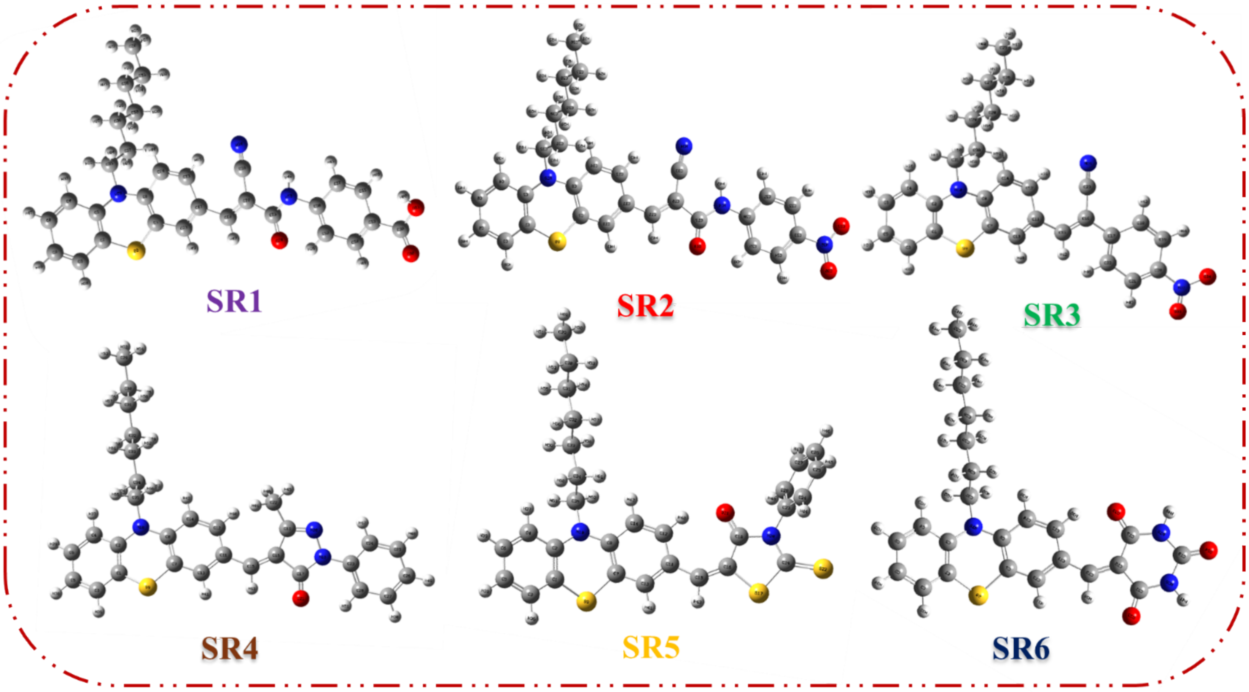

Figure (S27): Optimized structure of a dye sensitizer **SR1-6**.

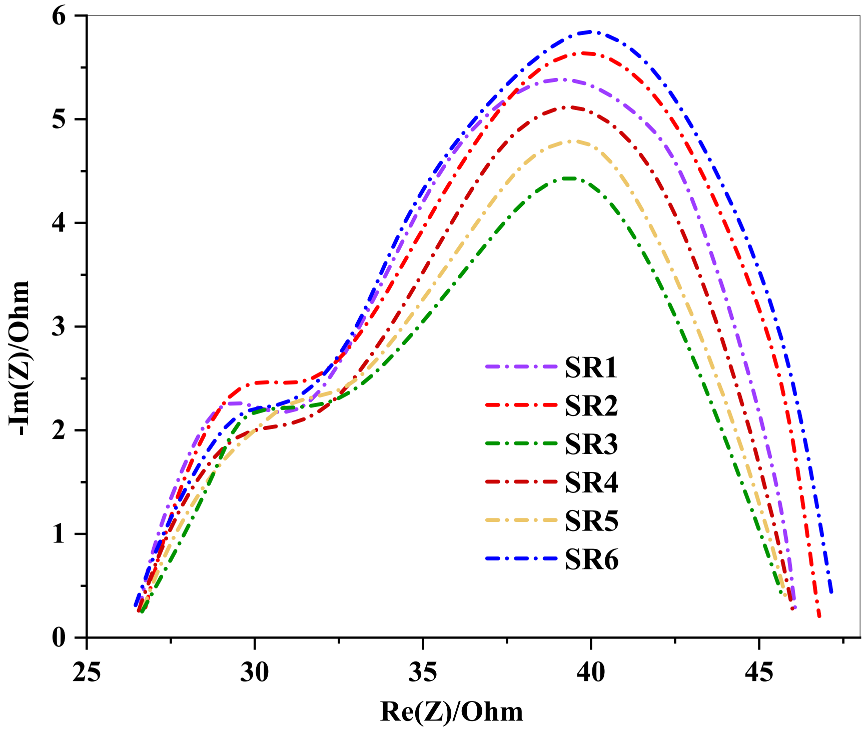

Figure (S28): Nyquist plots of SR1-6 based devices.

Fabrication process of sensitizers and co-sensitizers.

a) Working Electrode (Photoanode-TiO_2 electrode)

The fabrication process of dye-sensitized solar cells began with the preparation of a TiO_2 electrode, which consisted of a double-layer structure with a thickness of 10 + 5 µm. The electrode had a 10 mm thick nano-porous layer and a 5 µm thick scattering layer, which were prepared using a previously reported method [1]. The working electrode is subjected to 15 minutes of sonication in a detergent solution, followed by washing with deionized (DI) water, acetone, and ethanol. The electrode is then treated with $TiCl_4$ (60 mM) at 90°C for 60 minutes, and subsequently washed with water and ethanol. A layer of nanoporous TiO_2 (12-14 µm thick) is printed on the electrode using a single 3M transparent tape. The thickness of the layer is adjusted by punching a hole with a 3/16 5.0 stick. A dispersing layer is printed on top of the TiO_2 layer using a single 3M transparent tape. The thickness of the layer is adjusted by punching a hole with a 1/4 6.0 stick. The TiO_2 used for the dispersing layer is R/SP. The printed layers are sintered at 350°C for 10 minutes and then at 500°C for 30 minutes. The electrode is then treated with $TiCl_4$ at 90°C for 60 minutes and washed with water and ethanol. The electrode is sintered at 500°C for 30 minutes. In the absence of light, the electrode is submerged in a dye solution for

a duration of 20 hours. The organic dye solution is comprised of 0.2 mM of each (**SR-1-6**) in a mixture of acetonitrile, tert-butanol, and DMSO (1:1:1) with addition 0.2 Mm N719. Meanwhile, the ruthenium dye solution (N719 Dye) consists of 0.2 mM of the dye dissolved in 9 mL solution of consisting of (3 ml) acetonitrile, (3ml) tert-butanol, and (3ml) by ratio (1:1:1). In the case of co-sensitization, the dye solution consists of a mixture of 0.2 mM of the co-sensitizers (**SR-1-6**) and 0.2 mM of the ruthenium dye (N719 dye) in a 9 mL solution consisting of (3 ml) acetonitrile, (3ml) tert-butanol, and (3ml) DMSO by ratio (1:1:1). The performance of the dye-sensitized solar cells was characterized by photovoltaic measurements of sealed cells were made by illuminating the cell through the conducting glass from the anode side with a solar simulator at AM 1.5 illuminations (light intensity: 100 Mw.cm^{-2})

The preparation of a counter electrode involves the following steps:

The electrode is washed with water, followed by a wash with a 0.1M HCl solution in ethanol (0.2 mL of concentrated HCl in 100 mL of ethanol). The electrode is then subjected to 10 minutes of sonication in an acetone bath. The electrode is dried at 400°C for 15 minutes. A layer of Pt-paste is printed on the electrode using a single 3M transparent tape. The thickness of the layer is adjusted by punching a hole with a 3/8 10.0 stick. The Pt-paste used is Platisol T/SP. The printed layer is cured at 450°C for 10 minutes.

[1] Gad, E. A., Kamar, E. M., & Mousa, M. A. (2020). Experimental and computational study on electronic and photovoltaic properties of chromen-2-one-based organic dyes used for dye-sensitized solar cells. *Egyptian Journal of Petroleum*, *29*(2), 203-209.

2.2. Fabrication of dye-sensitized solar cell

Photovoltaic and incident photon-to-current efficiency (IPCE) measurements were made on sandwich cells, which were prepared using TiO_2 coated working electrodes and platinum coated counter electrodes and were sealed using a 40 μm Syrlyn spacer through heating of the polymer frame. The redox electrolyte (Solaronix, Iodolyte HI-30) consisted of a solution of 0.6 M DMPII, 0.05 M I_2, 0.1 M LiI and 0.5 M TBP in acetonitrile.

2.3. Photovoltaic measurements

Photovoltaic measurements of sealed cells were made by illuminating the cell through the conducting glass from the anode side with a solar simulator (WXS-155S-10) at AM 1.5 illuminations (light intensity: 100 mW cm^{-2}).

2.4. Incident photon to current efficiency (IPCE) conversion

IPCE measurements were made on a CEP-2000 system (Bunkoh-Keiki Co. Ltd.). IPCE at each wavelength was calculated using Equation 1, where I_{SC} is the short-circuit photocurrent density (mA. cm^{-2}) under monochromatic irradiation, q is the elementary charge, λ is the wavelength of incident radiation in nm and P0 is the incident radiative flux in W/m^2.

$$IPCE(\lambda) = 1240\left(\frac{I_{SC}}{q\lambda P_o}\right) \qquad (1)$$

2.4. Electrochemical impedance spectroscopy (EIS).

The electrochemical impedance spectra were measured with an impedance analyzer potentiostat (Bio-Logic SP-150) under illumination using a solar simulator (SOL3A, Oriel) equipped with a 450 W xenon lamp (91160, Oriel). EIS spectra were recorded over a frequency range of 100 mHz to 200 kHz at room temperature. The applied bias voltage was set at the V_{OC} of the DSSCs, with AC amplitude set at 10 mV. The electrical impedance spectra were fitted using Z-Fit software (Bio-Logic).

2.5. Cyclic voltammetry

Cyclic voltammetry (CV) was performed in DMF with the electrolyte 0.1 M [TBA][PF_6] at a scan rate of 100 mV s^{-1}. The working electrode used is the Glassy carbon, Pt wire represented the counter electrode and the reference electrode is Ag/Ag^+ in ACN. Fc/Fc^+ was introduced as internal reference. The CV measurements were performed on an Ivium Vertex electrochemical workstation by using the three electrode system.

3. Molecular Modeling

Equilibrium molecular geometries of **SR-1-6** calculated using the Becke's three parameter hybrid functional, Lee–Yang–Parr's gradient corrected correlation functional (B3LYP) and (6-311g(d, p)) [1, 2, 3, 4]. The geometry optimization calculations were followed by energy calculations using time-dependent density functional theory (TD-DFT) utilizing the energy, functional B3lyp and the basis set 6-311g (d, p). The solvent (DMF) effect was accounted for by using the conductor-like polarizable continuum model (C-PCM), implemented in Gaussian 09.

References

[1] G. Melikian, F. Rouessac, C. Alexandre, Synth Commun. 1993, 23, 2631
[2] A. D. Becke, Phys. Rev. A 1988, 38, 3098.
[3]C. T. Lee, W.T. Yang, R.G. Parr, Phys. Rev. B. 1988, 37, 785
[4] N. Godbout, D.R. Salahub, J. Andzelm, E. Wimmer, Optimization of Gaussian-type basis-sets for local spin-density functional calculations .1. Boron through neon, optimization technique and validation. Can. J. Chem.-Rev. Can. Chim. 1992, 70, 560-571.
